# Development and validation of a machine learning–based model for diagnosing perioperative malnutrition in older adults with hip fracture

**DOI:** 10.3389/fnut.2026.1751993

**Published:** 2026-06-08

**Authors:** Zhiqiang He, Mengyu Han, Yu An, Hui Xiao, Yaru Yang, Junxia Ye, Tianyu Wang, Jin Li

**Affiliations:** 1 School of Nursing, Health Science Center, Xi'an Jiaotong University, Xi'an, China; 2 Department of Nursing, Sun Yat-sen Memorial Hospital, Sun Yat-sen University, Guangzhou, China; 3 School of Nursing, Medical School of Yan'an University, Yan'an, China

**Keywords:** diagnostic model, hip fractures, influencing factors, malnutrition, older adults

## Abstract

**Background:**

The prevalence of malnutrition is significant among older adults with hip fractures, while existing screening tools face challenges such as complex procedures and a limited ability to objectively classify malnutrition status. This study aimed to develop and test a machine learning–based diagnostic model for identifying malnutrition guided by the Global Leadership Initiative on Malnutrition (GLIM) criteria.

**Methods:**

A cross-sectional study was conducted, enrolling patients from four tertiary hospitals in Xi'an between January and September 2024. Feature selection was performed using the Boruta and least absolute shrinkage and selection operator (LASSO) methods. Diagnostic classification models were constructed using five machine learning (ML) algorithms: logistic regression (LR), random forest (RF), support vector machine (SVM), extreme gradient boosting (XGBoost), and artificial neural network (ANN). Model performance was evaluated through receiver operating characteristic (ROC) analysis, decision curve analysis (DCA), and calibration curves. Shapley additive explanation (SHAP) values were applied for model interpretation. A total of 526 patients were ultimately included (385 in the training set and 141 in the external validation set), meeting the sample size requirements.

**Results:**

The prevalence of malnutrition among older adults with hip fractures was 38.78%. The key factors associated with malnutrition were age, body mass index (BMI), decreased appetite, chronic obstructive pulmonary disease (COPD), age-adjusted Charlson Comorbidity Index (aCCI), depression, albumin (ALB), and American Society of Anesthesiologists (ASA) classification, with the aCCI showing the greatest feature importance. The areas under the ROC curve (AUCs) for internal and external validation ranged from 0.8605 to 0.9424 and 0.8353 to 0.8565, respectively. All models except the ANN demonstrated good calibration, and DCA confirmed clinical usefulness across all models. The LR and XGBoost models demonstrated the best overall discriminative performance. Based on the LR model, an online calculator was developed and is accessible at: https://hip-fracture-malnutrition-test.shinyapps.io/dynnomapp/.

**Conclusion:**

This study employed ML to systematically assess the status and associated factors of perioperative malnutrition in elderly patients with hip fractures and developed quantifiable prediction models. The models demonstrated robust performance in both internal and external validation and were visualized *via* an online tool, providing practical guidance for early identification of malnourished patients and individualized nutritional interventions.

## 1 Introduction

Hip fracture is one of the most common and serious fragility fractures among older adults, characterized by a high incidence, substantial functional decline, and an elevated risk of mortality (1, 2). The global burden is projected to reach 6.3 million cases by 2050, with nearly 45% expected to occur in Asia (3). In China, the age-standardized incidence of hip fracture among older adults exceeds 110 per 100,000 person-years, accounting for over 1 million new cases annually (4, 5). Surgical intervention has become the standard treatment for hip fractures (6). However, prolonged postoperative bed rest often results in impaired gastrointestinal function, insufficient nutrient intake, and increased metabolic demands, leading to nutritional deterioration (7). Approximately 30%−60% of older patients with malnutrition or nutritional deficits during hospitalization (8). Malnutrition has been associated with frailty (9), delayed wound healing (10), and pulmonary infections (11), and it significantly increases the likelihood of prolonged hospital stay (12), secondary fractures (13), functional decline (14), and mortality (15). Therefore, early and accurate identification of malnutrition in older hip fracture patients is essential to optimize the effectiveness of nutritional interventions and improve clinical outcomes.

In recent years, several screening tools, such as the Nutritional Risk Score 2002 (NRS-2002) and the Mini Nutritional Assessment–Short Form (MNA-SF), have been widely applied to assess the status of malnutrition in older adults with hip fractures (16–19). However, these scales are primarily designed to identify individuals at nutritional risk and may not accurately diagnose the true disease status of malnutrition. The Global Leadership Initiative on Malnutrition (GLIM) is the standard tool for malnutrition assessment and recommends a two-step approach: first, using existing screening tools (e.g., NRS-2002, MNA-SF) to identify at-risk patients, followed by the GLIM criteria to confirm and grade malnutrition (20). Nevertheless, several limitations persist in clinical practice: (1) current tools do not provide quantitative estimates of malnutrition probability; (2) no screening tool has been specifically developed for older adults with hip fractures; (3) some key indicators are easily influenced by comorbid or pathological conditions—for example, body mass index (BMI) may be affected by tumors, ascites, or hematoma; and (4) certain measurements require complex procedures, specialized instruments, and substantial costs, making them impractical for use in general hospital wards. Therefore, there is an urgent need to develop a diagnostic screening tool that combines applicability, sensitivity, accuracy, and operational simplicity to meet the requirements of clinical malnutrition assessment.

Previous studies have shown that the presence of malnutrition in older adults with hip fractures is influenced by multiple complex factors, including demographic characteristics, disease- and treatment-related variables, biochemical parameters, and patient-reported symptoms (21–23). These multifactorial interactions make accurate clinical identification of malnutrition challenging. With the accumulation of large-scale medical data and advances in computational technologies, machine learning (ML) algorithms have shown great potential in medical research, particularly for risk prediction and clinical decision-making. However, most existing studies on malnutrition prediction have focused on patients with chronic wasting diseases, such as heart failure (24), hemodialysis (25), and cancer (26), while research targeting older adults with hip fractures remains limited. In addition, many published models are constrained by small sample sizes, lack of external validation, and relatively uniform modeling strategies (27). Owing to variation among assessment tools, the factors incorporated across studies also differ considerably, hindering the establishment of standardized clinical recommendations. Therefore, there is a pressing need to develop robust and generalizable diagnostic models based on established diagnostic frameworks to support the accurate identification and management of malnutrition in this vulnerable population.

In summary, malnutrition is a critical determinant of survival and postoperative outcomes in older adults with hip fractures; however, diagnostic-model research in this area remains at an early stage. In the present multicenter study, perioperative nutritional status and its associated factors were evaluated according to the GLIM criteria, and malnutrition diagnostic models were developed using five machine learning algorithms. The optimal model—identified *via* both internal and external validation—demonstrated robust discriminative performance and favorable clinical utility. These findings provide a practical and evidence-based tool to identify malnourished individuals, enable early detection and timely intervention, and support the development of personalized nutritional management strategies.

## 2 Materials and methods

### 2.1 Participant characteristics

This multicenter cross-sectional study was registered in the Chinese Clinical Trial Registry (ChiCTR2400082868) and included older adults with hip fractures. A total of 526 consecutive patients were recruited from four tertiary hospitals in Xi'an, Shaanxi Province, between January and September 2024. Eligible participants were consecutively enrolled to ensure representativeness. Inclusion criteria were: (1) age ≥ 60 years; (2) radiologically confirmed femoral neck, intertrochanteric, or subtrochanteric fracture; (3) scheduled for surgical treatment; (4) intact cognitive function with the ability to communicate and complete assessments; and (5) provision of written informed consent. Exclusion criteria were: (1) open or pathological fractures; (2) fractures resulting from high-energy trauma (e.g., traffic accidents, falls from height); (3) American Society of Anesthesiologists (ASA) class > IV or severe comorbidities such as hepatic or renal failure, systemic infection, or advanced malignancy; and (4) multiple organ dysfunction, severe arrhythmia, shock, or death during hospitalization.

### 2.2 Determination of sample size

The minimum required sample size for model development was calculated using the pmsampsize package in R (28). In this study, the outcome variable was binary, and the following parameters were entered: type = “b”, cstatistic = 0.87 (27), parameters = 10, prevalence = 0.30–0.60, yielding an internal validation sample size of 323–369 cases. For the external validation set, Riley et al. (28) suggested that at least 100 samples are required to ensure stable calibration assessment. Therefore, the total sample size was preliminarily determined to be 500, comprising 370 participants in the training set and 130 in the validation set. Finally, a total of 526 older adults with hip fractures were included, comprising 385 in the training set and 141 in the external validation set, indicating that the actual sample size met the research requirements.

### 2.3 Data collection and assessment instruments

A self-designed clinical data extraction form was developed to collect perioperative factors associated with malnutrition in older adults with hip fractures. All data were collected within 48 h of hospital admission and prior to surgery. It included six primary domains: general patient information, disease-related factors, blood biochemical parameters, surgery-related factors, medication use, and dietary-related factors, including a total of 91 secondary indicators. Data were obtained through a combination of face-to-face bedside interviews, review of electronic medical records, and biological measurements. Specific assessment instruments were as follows: the International Physical Activity Questionnaire-Short Form (IPAQ-SF) was used to quantify daily physical activity levels; the Barthel Index (BI) assessed functional independence in activities of daily living; the Athens Insomnia Scale (AIS) was used to evaluate the severity of insomnia symptoms over the past month; and the Patient Health Questionnaire-9 (PHQ-9) assessed depressive symptom severity during the previous 2 weeks. Oral health status was evaluated using the Oral Health Assessment Tool (OHAT), which was widely used in older adults and long-term care residents. Muscle mass was evaluated as follows: hand grip strength (HGS) was measured with an electronic dynamometer; calf circumference (CC) and mid-upper arm circumference (MUAC) were measured using a non-stretch tape measure with 0.1 cm graduations; triceps skinfold (TSF) thickness was measured with an electronic digital caliper to the nearest 0.1 mm. The mid-arm muscle circumference (MAMC) was calculated using the following formula: MAMC = MUAC – π × TSF.The GLIM diagnostic framework and the cut-off values for muscle quality assessment indicators are detailed in Table S1.

### 2.4 Development of machine learning diagnostic models

Least absolute shrinkage and selection operator (LASSO) regression was performed using the glmnet package in R to identify features with significant associative value for the target variable (i.e., malnutrition status). Features with non-zero coefficients from the LASSO regression were retained as independent variables, with malnutrition status serving as the binary dependent variable. Subsequently, backward stepwise logistic regression was conducted using the MASS package in R to minimize the Akaike Information Criterion (AIC) and further refine variable selection. The final set of variables was determined by integrating these statistical results with clinical expertise. Building on these results, five commonly used machine learning algorithms for classification were applied: LR, RF, SVM, XGBoost, and ANN. The LR model was constructed using backward elimination with all candidate features, implemented in R with the glm and step functions. The RF, SVM, XGBoost, and ANN models were developed using the randomForest, e1071, xgboost, and nnet packages, respectively. Hyperparameters were optimized using a grid search strategy within the training set. In addition, five-fold cross-validation was performed to assess model robustness and provide a less biased estimate of predictive performance.

### 2.5 Model validation and evaluation

A total of 385 patients from three tertiary hospitals, excluding the Second Affiliated Hospital of Xi'an Jiaotong University, were assigned to the training set for model development. To evaluate the reliability and stability of the model, internal validation was performed using the bootstrap method, implemented in R with the boot package, with 500 bootstrap resamples. The dataset was divided into a training set and an external validation set based on the source of the samples. Specifically, 385 patients from the other three hospitals constituted the training set for model development, and 141 patients from the Second Affiliated Hospital of Xi'an Jiaotong University were assigned to the external validation set. The external validation set was entirely independent of the training set, ensuring the objectivity and reliability of the test results and providing a more accurate assessment of model performance in real-world clinical settings. However, it should be noted that all participating hospitals were located within the same geographic region (Xi'an), which may limit the heterogeneity of the external validation cohort. The discriminatory performance of the models was evaluated using receiver operating characteristic (ROC) curves and the area under the curve (AUC). Model calibration was assessed through calibration plots, the Hosmer–Lemeshow goodness-of-fit test, and the Brier score.

### 2.6 Clinical utility assessment

DCA was performed to evaluate the clinical utility of the nomogram in practical application. The clinical net benefit of the nomogram was assessed separately in the training set, validation set, and overall population. The DCA was conducted in R using the Tableone and Nonrandom packages.

### 2.7 Statistical analysis

Data entry was performed using Microsoft Excel 2019, and statistical analyses were conducted with SPSS version 26.0, R version 4.4.2 (within the R Studio environment), and MedCalc version 20.0.4. Missing data were managed using multiple imputation (MI) techniques. Among the 91 candidate variables, only six variables [CRP, 25-(OH)VD3, TC, TG, HDL, LDL] had missing values, ranging from 14.64 to 85.82%, while all other variables were complete (see Table S2). The missingness mechanism was assumed to be Missing at Random (MAR) based on clinical plausibility and exploratory data patterns. Multiple imputation by chained equations (MICE) was applied using the R package mice, generating 10 imputed datasets with 20 iterations each. Convergence was evaluated through trace plots and parameter stability checks. To avoid data leakage, imputation was performed separately for the training (*n* = 385) and external validation (*n* = 141) datasets. Pooled estimates across imputed datasets were calculated using Rubin's rules. The normality of continuous variables was assessed using the Shapiro–Wilk test. Normally distributed continuous variables are presented as mean ± standard deviation (SD), while non-normally distributed variables are expressed as median and interquartile range [M (P25, P75)].

## 3 Results

### 3.1 Baseline characteristics of participants

From January to September 2024, data were collected from the orthopedics and trauma surgery departments of four tertiary Grade A general hospitals in Xi'an, Shaanxi Province, in accordance with strict inclusion and exclusion criteria. During the questionnaire collection process, 554 elderly patients with hip fractures were initially recruited. Of these, 28 patients were excluded due to reasons including non-surgical treatment, transfer to another hospital, or in-hospital mortality. Ultimately, 526 valid questionnaires were retained, yielding an effective response rate of 94.95%. Of the total sample, 204 patients (38.78%) were identified as malnourished. The mean age of the total sample was 75.40 ± 8.84 years, with a range from 60 to 97 years. The mean BMI was 22.51 ± 3.37 kg/m^2^. Females constituted the majority (66.54%) of the cohort. Educational attainment was predominantly at the primary school level or below (30.04%) and junior high school level (33.84%). The most common range of monthly per capita household income was 3,000–5,000 RMB (59.70%). Prior to admission, the majority of patients (64.45%) lived with their spouse. Most patients reported no history of smoking (84.41%) or alcohol consumption (88.78%). Physical activity levels were predominantly moderate (54.94%), while only a minority (2.47%) reported high activity levels. The distributions of other demographic characteristics are detailed in Table 1.

**Table 1:** Comparison of differences in patient demographic characteristics between the training and validation sets.

Variables	Overall (*N* = 526)	Training set (*N* = 385)	External validation set (*N* = 141)	Statistics	*P* value
Age	75.40 ± 8.84	75.27 ± 8.78	75.77 ± 9.04	*t* = −0.57	0.57
Sex				χ^2^ = 0.64	0.43
Male	176 (33.46)	125 (32.47)	51 (36.17)		
Female	350 (66.54)	260 (67.53)	90 (63.83)		
BMI (kg/m^2^)	22.51 ± 3.37	22.51 ± 3.20	22.51 ± 3.81	*t* = −0.01	0.10
Education level				χ^2^ = 8.59	0.04
Primary school or below	158 (30.04)	121 (31.43)	37 (26.24)		
Junior high school	178 (33.84)	139 (36.10)	39 (27.66)		
High school or secondary specialized school	125 (23.76)	81 (21.04)	44 (31.21)		
College or above	65 (12.36)	44 (11.43)	21 (14.89)		
Monthly household income per capita (yuan)				χ^2^ = 0.16	0.92
<3000	83 (15.78)	62 (16.10)	21 (14.89)		
3000~5000	314 (59.70)	228 (59.22)	86 (60.99)		
>5000	129 (24.52)	95 (24.68)	34 (24.11)		
Pre-admission living arrangement				χ^2^ = 1.12	0.57
Living with spouse	339 (64.45)	245 (63.64)	94 (66.67)		
Living with children	160 (30.42)	118 (30.65)	42 (29.79)		
Living alone or in a nursing institution	27 (5.13)	22 (5.71)	5 (3.55)		
Smoking history				χ^2^ = 0.00	0.10
No	444 (84.41)	325 (84.42)	119 (84.40)		
Yes	82 (15.59)	60 (15.58)	22 (15.60)		
Drinking history				χ^2^ = 0.14	0.71
No	467 (88.78)	343 (89.09)	124 (87.94)		
Yes	59 (11.22)	42 (10.91)	17 (12.06)		
Physical activity level				-	0.02
Low	224 (42.59)	165 (42.86)	59 (41.84)		
Moderate	289 (54.94)	215 (55.84)	74 (52.48)		
High	13 (2.47)	5 (1.30)	8 (5.67)		
Pain score	2 (2, 2)	2 (2, 2)	2 (1, 2)	*Z* = −1.60	0.11
Decreased appetite				χ^2^ = 0.15	0.70
No	376 (71.48)	277 (71.95)	99 (70.21)		
Yes	150 (28.52)	108 (28.05)	42 (29.79)		
Activities of daily living, ADL (BI)	35 (25, 45)	35 (25, 45)	40 (30, 50)	*Z* = −2.54	0.01
Urination status				-	0.04
Normal	510 (96.96)	377 (97.92)	133 (94.33)		
Abnormal	16 (3.04)	8 (2.08)	8 (5.67)		
Defecation status				χ^2^ = 12.75	<0.001
Normal	326 (61.98)	221 (57.40)	105 (74.47)		
Abnormal	200 (38.02)	164 (42.60)	36 (25.53)		

Mean +/- SD; Median (P25, P75); n (%); Student's t-test (t); chi-square test (χ^2^); Mann-Whitney U test (Z); Fisher's exact test (-).

The prevalence of malnutrition was 38.44% (148 cases) in the training set and 39.72% (56 cases) in the validation set. Univariate analyses were conducted to compare the training and validation sets with respect to demographic characteristics, disease-related factors, blood biochemical parameters, surgery-related factors, medication use, dietary factors, and nutritional assessment indicators. The results indicated that significant differences (*P* < 0.05) were present between the two sets in the distributions of educational level, physical activity level, self-care ability, urinary continence, and defecation status. Other demographic variables did not differ significantly (Table 1). Regarding disease-related factors, only the distribution of fracture types differed significantly between the sets (*P* < 0.05), while the remaining variables showed no significant differences (Table S3). For blood biochemical parameters, significant differences were observed for PLR (Platelet-to-Lymphocyte Ratio), PA (Prealbumin), Ca^2+^ (Calcium), Cr (Creatinine), and AST (Aspartate Aminotransferase; *P* < 0.05), with other parameters showing no significant variation (Table S4).

In surgery-related factors, significant differences were detected in operation duration, anesthesia duration, postoperative drainage volume, and postoperative bed-rest duration (*P* < 0.05), whereas other variables did not differ significantly (Table S5). For medication use and dietary factors, significant differences were observed in the distributions of diuretics, acid-suppressing or gastroprotective agents, and daily dietary fiber intake between the training and validation sets (*P* < 0.05), with no significant differences observed in other factors (Table S6). Concerning nutritional assessment indicators, no significant differences (*P* > 0.05) were found in any nutritional parameter or malnutrition status between the training and validation sets (Table S7).

### 3.2 Univariate analysis

Independent *t*-tests were applied to age and BMI. Mann–Whitney U tests were applied to the remaining continuous variables. Fisher's exact test was applied to physical activity level and urinary continence. Eleven demographic characteristics were significantly associated (*P* < 0.05) with malnutrition in patients with hip fractures: age, BMI, educational level, monthly household income per capita, pre-admission living arrangement, physical activity level, pain score, decreased appetite, BI, urinary continence, and defecation status (see Table 2 for details). Regarding disease-related factors, 16 variables showed significant associations (*P* < 0.05) with malnutrition: myocardial infarction/coronary heart disease, congestive heart failure, peripheral vascular disease, cerebrovascular disease, dementia/Alzheimer's disease, COPD, solid tumor, moderate-to-severe chronic kidney disease, hypertension, visual impairment, aCCI, sleep disturbance, depression, oral health, swallowing function, and time from fracture to surgery (see Table S8). Fourteen blood biochemical parameters were significantly associated (*P* < 0.05) with malnutrition: PA, Ca^2+^, PLR, HGB (Hemoglobin), WBC (White Blood Cell count), LYM (Lymphocyte count), NEUT (Neutrophil count), PLT (Platelet count), NLR (Neutrophil-to-Lymphocyte Ratio), CRP (C-Reactive Protein), TP (Total Protein), ALB (Albumin), BUN (Blood Urea Nitrogen), and ALT (Alanine Aminotransferase; see Table S9). Six surgery-related factors were significantly associated (*P* < 0.05) with malnutrition: surgical approach, intraoperative blood transfusion, ASA classification, preoperative fasting duration for clear fluids, postoperative drainage volume, and postoperative bed-rest duration (see Table S10). Ten medication use and dietary factors showed significant associations (*P* < 0.05) with malnutrition: hypoglycemic agents, diuretics, corticosteroids, acid-suppressing/gastroprotective agents, antipsychotics, daily protein intake, daily carbohydrate intake, daily dietary fiber intake, daily fat intake, and total daily energy intake (see Table S11).

**Table 2:** Univariate analysis of demographic characteristics for malnutrition in the training set.

Variables	Training set (*N* = 385)	Well-nourished (*N* = 237)	Malnourished (*N* = 148)	Statistics	*P* value
Age	75.27 ± 8.78	71.89 ± 7.80	80.68 ± 7.47	*t* = −10.94	<0.001
Sex				χ^2^ = 0.82	0.37
Male	125 (32.47)	81 (34.18)	44 (29.73)		
Female	260 (67.53)	156 (65.82)	104 (70.27)		
BMI (kg/m^2^)	22.51 ± 3.20	23.05 ± 2.84	21.63 ± 3.54	*t* = 4.11	<0.001
Education level				χ^2^ = 15.11	0.002
Primary school or below	121 (31.43)	58 (24.47)	63 (42.57)		
Junior high school	139 (36.10)	94 (39.66)	45 (30.41)		
High school or secondary specialized school	81 (21.04)	58 (24.47)	23 (15.54)		
College or above	44 (11.43)	27 (11.39)	17 (11.49)		
Monthly household income per capita (yuan)				χ^2^ = 9.66	0.008
<3000	62 (16.10)	28 (11.81)	34 (22.97)		
3000~5000	228 (59.22)	152 (64.14)	76 (51.35)		
>5000	95 (24.68)	57 (24.05)	38 (25.68)		
Pre-admission living arrangement				χ^2^ = 30.53	<0.001
Living with spouse	245 (63.64)	176 (74.26)	69 (46.62)		
Living with children	118 (30.65)	50 (21.10)	68 (45.95)		
Living alone or in a nursing institution	22 (5.71)	11 (4.64)	11 (7.43)		
Smoking history				χ^2^ = 3.07	0.08
No	325 (84.42)	194 (81.86)	131 (88.51)		
Yes	60 (15.58)	43 (18.14)	17 (11.49)		
Drinking history				χ^2^ = 1.12	0.29
No	343 (89.09)	208 (87.76)	135 (91.22)		
Yes	42 (10.91)	29 (12.24)	13 (8.78)		
Physical activity level				-	<0.001
Low	165 (42.86)	55 (23.21)	110 (74.32)		
Moderate	215 (55.84)	177 (74.68)	38 (25.68)		
High	5 (1.30)	5 (2.11)	0 (0.00)		
Pain score	2 (2, 2)	2 (2, 2)	2 (2, 2)	*Z* = −2.71	0.01
Decreased appetite				χ^2^ = 72.39	<0.001
No	277 (71.95)	207 (87.34)	70 (47.30)		
Yes	108 (28.05)	30 (12.66)	78 (52.70)		
Activities of daily living, ADL (BI)	35 (25, 45)	40 (30, 55)	30 (25, 35)	*Z* = −7.46	<0.001
Urination status				-	0.01
Normal	377 (97.92)	236 (99.58)	141 (95.27)		
Abnormal	8 (2.08)	1 (0.42)	7 (4.73)		
Defecation status				χ^2^ = 17.88	<0.001
Normal	221 (57.40)	156 (65.82)	65 (43.92)		
Abnormal	164 (42.60)	81 (34.18)	83 (56.08)		

Mean +/- SD; Median (P25, P75); n (%); Student's t-test (t); chi-square test (χ^2^); Mann-Whitney U test (Z); Fisher's exact test (-).

**Figure 1:**
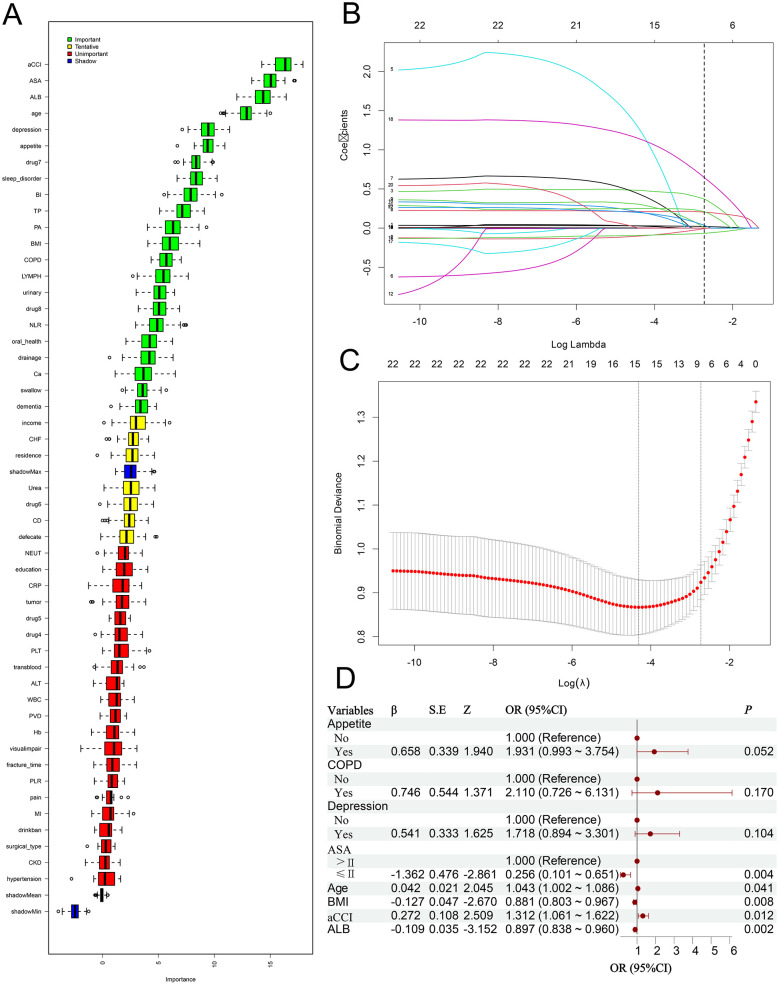
Feature selection and multivariable modeling. **(A)** Importance ranking of clinical variables using the Boruta algorithm. Green boxplots indicate confirmed important attributes, yellow indicate tentative attributes, red indicate unimportant attributes, and blue represent shadow attributes. The y-axis shows variable names, and the x-axis shows Z-scores. **(B)** LASSO coefficient path plot. The trajectories show how variable coefficients change with the penalty parameter (λ). As λ increases, some coefficients shrink toward zero. The y-axis represents standardized coefficients, the x-axis shows the log(λ), and the upper x-axis indicates the number of predictors retained. **(C)** Cross-validation error curve. Ten-fold cross-validated binomial deviance is plotted against log(λ). The two vertical dashed lines represent the log(λ) corresponding to the minimum model error (λ_min) and the most regularized model within one standard error of the minimum error (λ_1se). The y-axis shows binomial deviance, and the x-axis shows log(λ), with the upper axis indicating the number of predictors. **(D)** Forest plot of the multivariable backward stepwise logistic regression. Odds ratios with 95% confidence intervals are displayed for each predictor retained in the final model.

Although univariate analysis suggested that dietary factors—including daily protein, carbohydrate, dietary fiber, fat, and total energy intake—might be associated with malnutrition, these five variables were excluded from the final model due to potential recall bias and inaccuracies in dietary reporting, which are particularly pronounced in older adults with cognitive decline. Subsequently, the Boruta algorithm was applied to the remaining 52 variables that were significant in the univariate analysis. Twenty-two variables were confirmed as important: age, BMI, decreased appetite, BI, urinary continence, dementia/Alzheimer's disease, COPD, aCCI, depression, sleep disturbance, oral health, swallowing function, LYM, NLR, TP, ALB, PA, Ca^2+^, ASA classification, postoperative drainage volume, acid-suppressing/gastroprotective agents, and antipsychotics. Twenty-three variables were confirmed as unimportant: educational level, physical activity level, pain score, myocardial infarction/coronary heart disease, peripheral vascular disease, solid tumor, moderate-to-severe chronic kidney disease, hypertension, visual impairment, time from fracture to surgery, postoperative bed-rest duration, HGB, WBC, NEUT, PLT, PLR, CRP, ALT, surgical approach, intraoperative blood transfusion, preoperative fasting duration for clear fluids, hypoglycemic agents, and diuretics. Seven variables remained tentative: monthly household income per capita, pre-admission living arrangement, defecation status, congestive heart failure, cerebrovascular disease or TIA, BUN, and corticosteroids. See Figure 1A for detailed results.

The 22 variables confirmed as important by the Boruta algorithm were subsequently included in the LASSO regression. The optimal regularization parameter (λ) was determined using 10-fold cross-validation. As shown in Figure 1B, the coefficients of certain variables approached zero as log(λ) increased. To balance a stringent shrinkage penalty with control over mean squared error (MSE), the λ value corresponding to one standard error above the minimum MSE (lambda_1se) was selected as the optimal parameter (Figure 1C), resulting in a final λ of 0.065. This process resulted in 10 variables retained with non-zero coefficients: age, BMI, decreased appetite, COPD, aCCI, depression, sleep disturbance, oral health, ALB, and ASA classification.

### 3.3 Multivariable analysis

The 10 candidate variables were entered as independent variables into a binary backward stepwise logistic regression analysis, which sequentially removed variables to identify the model with the smallest AIC (29). Table S12 shows that after removing sleep disturbance and oral health, the final model achieved a minimum AIC value of 326.82. Table 3 presents the regression coefficients, significance test results, and odds ratios (OR) with 95% confidence intervals (95% CI) for each variable. The *P*-values of decreased appetite, COPD, and depression exceeded 0.05, whereas the remaining variables were statistically significant (*P* < 0.05). Based on expert consultation and the minimum AIC criterion, these three variables were retained in the model. Consequently, eight variables were ultimately included in the diagnostic model: age, BMI, decreased appetite, COPD, aCCI, depression, ALB, and ASA classification. BMI and ALB were identified as protective factors (OR <1), indicating that higher values were associated with a lower likelihood of concurrent malnutrition, while the remaining variables were factors positively associated with malnutrition status. Figure 1D presents a forest plot of the logistic regression results.

**Table 3:** Backward stepwise logistic regression analysis of factors associated with malnutrition in older adults with hip fracture.

Variables	Coefficient	SE	*Z* value	*P* value	OR (95% CI)
Constant	0.28	2.28	0.12	0.90	1.32 (0.02–114.43)
Age	0.04	0.02	2.05	0.04	1.04 (1.00–1.09)
BMI (kg/m^2^)	−0.13	0.05	−2.67	0.01	0.88 (0.80–0.97)
aCCI	0.27	0.11	2.51	0.01	1.31 (1.06–1.62)
ALB (g/L)	−0.11	0.04	−3.15	0.002	0.90 (0.84–0.96)
Decreased appetite					
No					1.00 (Reference)
Yes	0.69	0.34	1.94	0.05	1.93 (0.99–3.75)
COPD					
No					1.00 (Reference)
Yes	0.75	0.54	1.37	0.170	2.11 (0.73–6.13)
Depression					
No					1.00 (Reference)
Yes	0.54	0.33	1.63	0.10	1.72 (0.89–3.30)
ASA					
≤ II					1.000 (Reference)
>II	1.36	0.48	2.86	0.004	3.91 (1.54–9.93)

OR, odds ratio; CI, confidence interval; aCCI, age-adjusted Charlson comorbidity index; ALB, albumin; COPD, chronic obstructive pulmonary disease; ASA, American society of anesthesiologists.

### 3.4 Model development and hyperparameter tuning

Feature selection, based on Boruta, LASSO, and backward stepwise logistic regression, identified eight core variables for model development: age, BMI, decreased appetite, COPD, aCCI, depression, ALB, and ASA classification. Hyperparameters were optimized using a 5-fold cross-validated grid search conducted exclusively on the training set, with AUC maximization as the optimization objective. The final hyperparameter configurations for the five algorithms were as follows: LR employed backward stepwise selection (family = binomial, direction = “backward”); RF used ntree = 50, mtry = 1, and nodesize = 5 with bootstrap sampling; SVM applied a radial basis function kernel with cost = 1 and gamma = 0.1; XGBoost was configured with nrounds = 100, eta = 0.1, max_depth = 2, subsample = 0.8, min_child_weight = 1, and colsample_bytree = 0.8; and ANN was set with size = 10 and maxit = 200. Complete hyperparameter specifications, search ranges, and tuning results for all five algorithms are provided in Supplementary Table S13.

### 3.5 Model performance evaluation

Based on the training set data, this study developed diagnostic models for malnutrition in older hip fracture patients using five ML algorithms: LR, RF, SVM, XGBoost, and ANN. The ROC curve analysis showed that the XGBoost model achieved the highest AUC (0.94, 95% CI: 0.92–0.96), followed by the RF model (0.94, 95% CI: 0.91–0.96). The AUC values of the remaining models, in descending order, were: ANN (0.90, 95% CI: 0.87–0.93), SVM (0.90, 95% CI: 0.86–0.93), and LR (0.89, 95% CI: 0.86–0.92). All models achieved AUC values close to or exceeding 0.9, indicating high predictive performance in the training set and an effective ability to discriminate patients with malnutrition.

**Figure 2:**
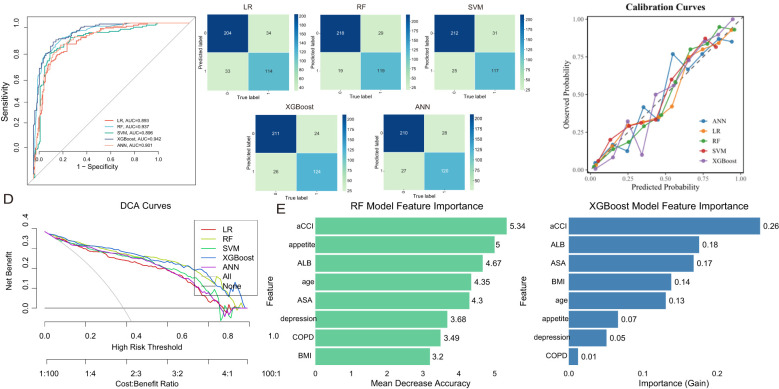
*Model performance in the training set*. **(A)** ROC curve for the training dataset. **(B)** Confusion matrix for the training dataset. Axis labels: 0 = normal nutrition; 1 = malnutrition. **(C)** Calibration curve for the training dataset. **(D)** Decision curve analysis (DCA) for the training dataset. **(E)** Variable importance ranking based on the final predictive model.

To provide a clear and comprehensive evaluation of classification performance, confusion matrices were generated for each model (Figure 2A). These matrices visually present the distribution of correctly and incorrectly classified samples, facilitating assessment of model performance. Based on the confusion matrices, key metrics—including sensitivity, specificity, accuracy, precision, and F1-score—were calculated for each model and subsequently normalized relative to the minimum observed values. The metrics were then normalized with reference to their minimum values, and a radar chart illustrating these normalized evaluation metrics from the training set confusion matrices was plotted (Figure 2B). The results revealed that the LR model demonstrated the weakest performance across all metrics. In contrast, other models showed distinct strengths across different metrics: the RF model achieved the highest sensitivity (0.86) and accuracy (0.88), whereas the XGBoost model attained the highest specificity (0.90) and precision (0.84). Furthermore, both the RF and XGBoost models attained an identical F1-score of 0.83, representing the highest value on this metric. These findings indicate that different models have specific strengths in the classification task, with XGBoost and RF models demonstrating robust overall performance across multiple metrics.

To evaluate the calibration performance of the diagnostic models, calibration curves were plotted for each model (Figure 2C). The results indicated that the calibration curves of the ANN and XGBoost models showed noticeable deviation from the reference line, whereas the curves of the other models closely aligned with the reference line, indicating satisfactory calibration.

Furthermore, DCA was performed to evaluate the clinical utility of the diagnostic models (Figure 2D). The results indicated that across a specific threshold probability range, the net benefits of all models consistently exceeded both the “All” (intervene for all) and “None” (intervene for none) reference lines, demonstrating clinical usefulness for all developed models. A further comparison revealed that the XGBoost model exhibited the widest applicable threshold range, approximately 0.02–0.98, followed by the RF model, whereas the ANN and SVM models had relatively narrower ranges. These findings suggest that the XGBoost model has broader applicability and greater potential for clinical decision-making.

**Figure 3:**
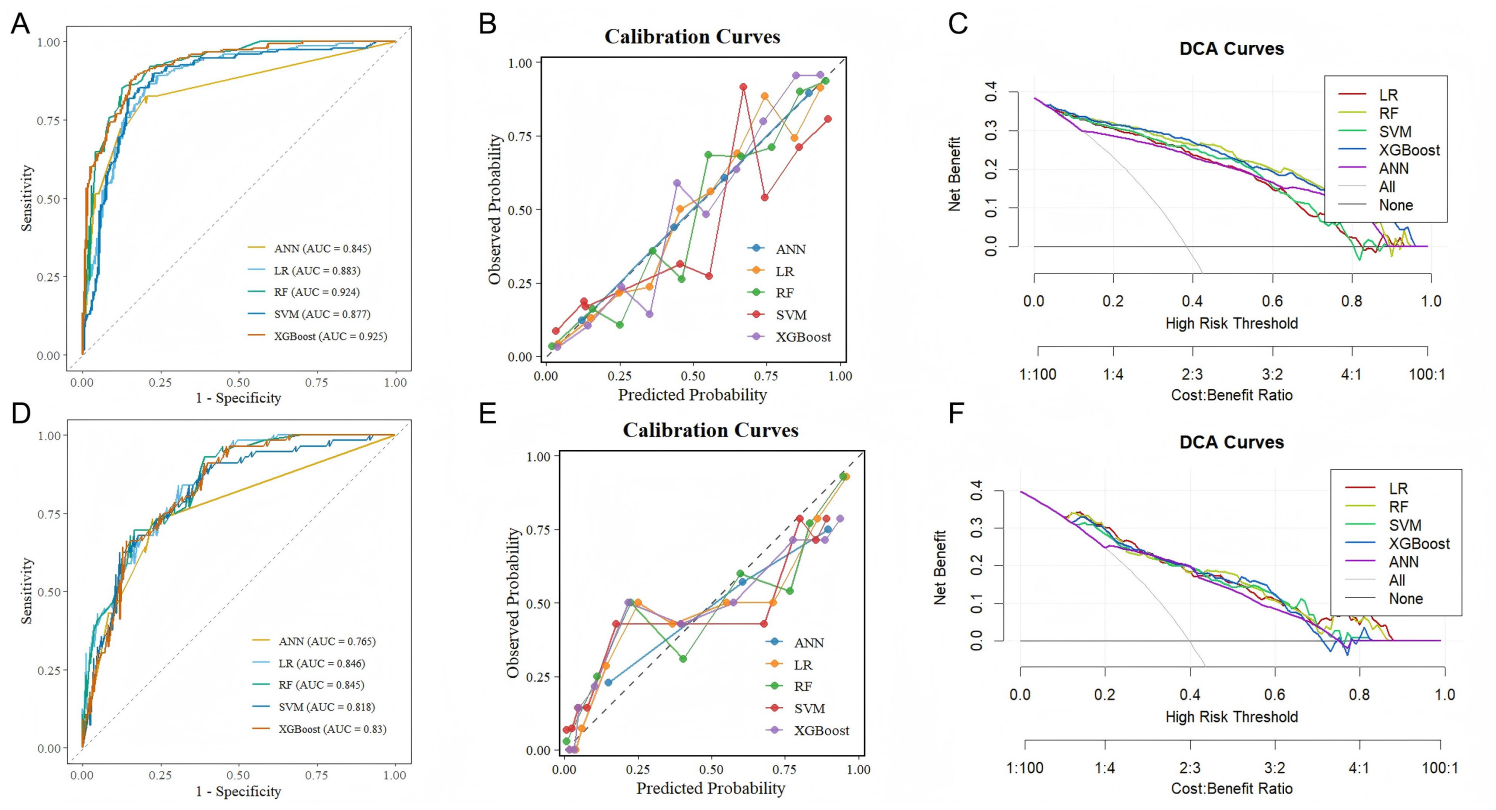
Model validation after removing BMI and decreased appetite. **(A)** ROC curve of internal training set after removing BMI and decreased appetite. **(B)** Calibration curve of internal training set after removing BMI and decreased appetite. **(C)** DCA curve of internal training set after removing BMI and decreased appetite. **(D)** ROC curve of external validation set after removing BMI and decreased appetite. **(E)** Calibration curve of external validation set after removing BMI and decreased appetite. **(F)** DCA curve of external validation set after removing BMI and decreased appetite.

To further assess the robustness of the model performance and evaluate the potential incorporation bias arising from the inclusion of GLIM-overlapping variables (BMI and decreased appetite), a sensitivity analysis was conducted. All five machine learning models were re-developed after excluding these two variables, using the remaining six predictors (age, aCCI, ALB, COPD, depression, and ASA classification). All modeling procedures, including hyperparameter tuning and validation strategies, remained unchanged. In the training set, the exclusion of these variables resulted in only minimal reductions in AUC across all models (ΔAUC: −0.01 to −0.06), with substantial overlap in 95% confidence intervals between the original and reduced models. Similarly, in the external validation set, AUC values decreased marginally (ΔAUC: −0.01 to −0.07), and most differences were not statistically significant. These findings indicate that the discriminative performance of the models was not primarily driven by the inclusion of GLIM-overlapping variables. Instead, other predictors, particularly ALB, ASA classification, age, and aCCI, contributed substantially to model performance (see Table S14-15). The ROC curves comparing the original and reduced models are presented in Figure 3.

Overall, the RF and XGBoost models demonstrated superior performance compared to the other models. Although the XGBoost model achieved a slightly higher AUC than the RF model (by 0.0059), statistical testing indicated that this difference was not significant (*P* = 0.22). Notably, the feature importance analysis was based on the original models including all predictors. To further explore the classification mechanisms and identify key features, feature importance analysis was performed on both the RF and XGBoost models to identify the core features contributing to model outputs. Figure 2E shows the feature importance ranking in the RF model based on Mean Decrease Accuracy (MDA), where the values reflect each feature's contribution to classification accuracy. The results indicate that aCCI (5.34), decreased appetite (5.00), and ALB (4.67) were the three most important features in the RF model. The remaining features were ranked in descending order of importance as follows: age (4.35), ASA classification (4.30), depression (3.68), COPD (3.49), and BMI (3.20). Figure 2F presents the feature importance ranking in the XGBoost model based on Gain values, which quantify each feature's contribution by its improvement in discriminative performance. Consistently, aCCI was the most important feature in the XGBoost model. The overall importance scores, in descending order, were: aCCI (0.26), ALB (0.18), ASA classification (0.17), BMI (0.14), age (0.13), decreased appetite (0.07), depression (0.05), and COPD (0.01). This ranking differs from that observed in the RF model, indicating differences in feature weighting between the two models. In summary, although both models identified aCCI as a key feature, the differences in importance scores and ranking order suggest that the internal mechanisms of RF and XGBoost interpret and utilize features in distinct ways.

### 3.6 Internal validation of the diagnostic model

**Figure 4:**
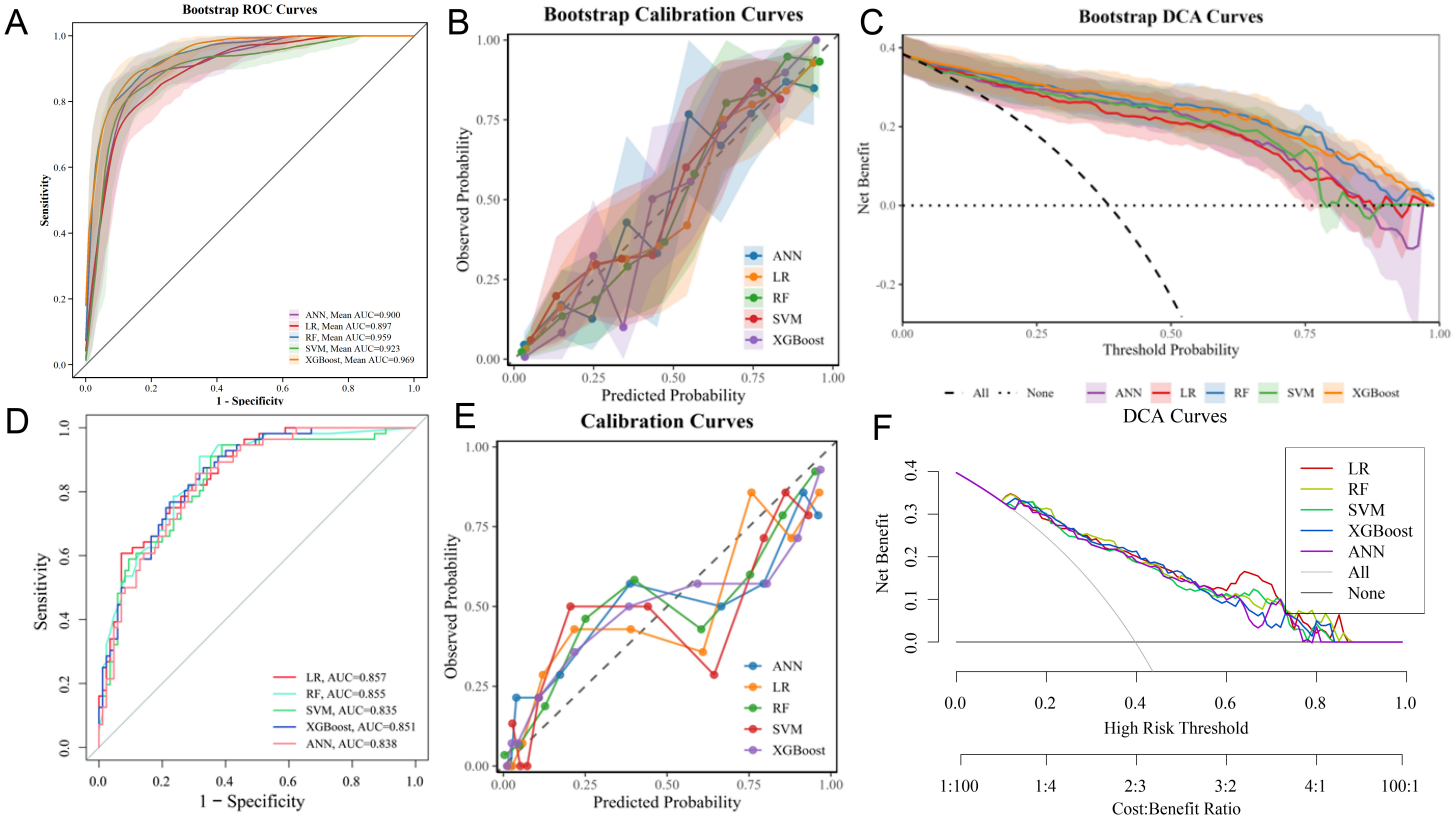
Internal and external validation performance. **(A)** ROC curve for the internal validation dataset. **(B)** Calibration curve for the internal validation dataset. **(C)** DCA curve for the internal validation dataset. **(D)** ROC curve for the external validation dataset. **(E)** Calibration curve for the external validation dataset. **(F)** DCA curve for the external validation dataset.

Internal validation was performed using 500 bootstrap resampling iterations within the training set. During internal validation, the XGBoost model maintained the highest mean AUC (0.97, 95% CI: 0.95–0.98), followed by the RF model (0.96, 95% CI: 0.94–0.98). The remaining models, ranked in descending order of mean AUC, were: SVM (0.92, 95% CI: 0.89–0.95), ANN (0.90, 95% CI: 0.87–0.93), and LR (0.90, 95% CI: 0.87–0.93; Figure 4A). All models achieved mean AUC values close to or exceeding 0.9, indicating high discriminative performance during internal validation. A comparison with the original training set performance revealed that, with the exception of the ANN model, which showed a slight decrease in AUC (by 0.0016), all other models showed performance similar to that in the training set.

The calibration evaluation results for all models during internal validation are presented in Figure 4B. Analysis of the calibration curves revealed that the LR, RF, and XGBoost models demonstrated good calibration performance.

Figure 4C illustrates the clinical utility evaluation of each model in the internal validation set. The results demonstrate that all models showed favorable clinical net benefit, as evidenced by net benefit curves substantially exceeding both the “All” and “None” reference lines across specific threshold probability ranges. Further comparison revealed that the RF and XGBoost models exhibited the widest applicable threshold ranges, followed by the LR and ANN models, while the SVM model showed the narrowest range—a finding highly consistent with the clinical utility results observed in the training set. These outcomes indicate that while all models possess clinical value, the XGBoost and RF models show broader applicability in clinical practice.

### 3.7 External validation of the diagnostic model

In the external validation set, the LR model achieved the highest AUC value (0.86, 95% CI: 0.80–0.92), followed closely by the RF model (0.86, 95% CI: 0.79–0.92). The remaining models demonstrated AUC values in descending order as follows: XGBoost (0.8508, 95% CI: 0.79–0.91), ANN (0.84, 95% CI: 0.77–0.90), and SVM (0.84, 95% CI: 0.77–0.90; Figure 4D). All models achieved AUC values ranging from 0.8 to 0.9, indicating acceptable discriminative performance in the external validation set and suggesting their potential suitability for clinical use.

The calibration evaluation results for all models in the external validation set are shown in Figure 4E. The calibration curves showed noticeable deviations from the ideal reference line across the models, indicating suboptimal calibration performance.

Figure 4F illustrates the clinical utility assessment of each model in the external validation set. As shown, all models demonstrated good clinical utility, with net benefit curves substantially exceeding both the “treat-all” and “treat-none” reference lines across specific threshold probability ranges. Further comparison revealed that the LR and RF models had the widest applicable threshold ranges, approximately 0.10–0.85, followed by the XGBoost and SVM models, while the ANN model exhibited the narrowest range. However, the differences among models were relatively modest. Compared with the training set, the DCA curves in the external validation set remained largely consistent, indicating that all models maintained robust stability and generalizability across different clinical settings, supporting their potential application in clinical decision-making for malnutrition malnutrition in older hip fracture patients.

### 3.8 Model visualization and interpretation

**Figure 5:**
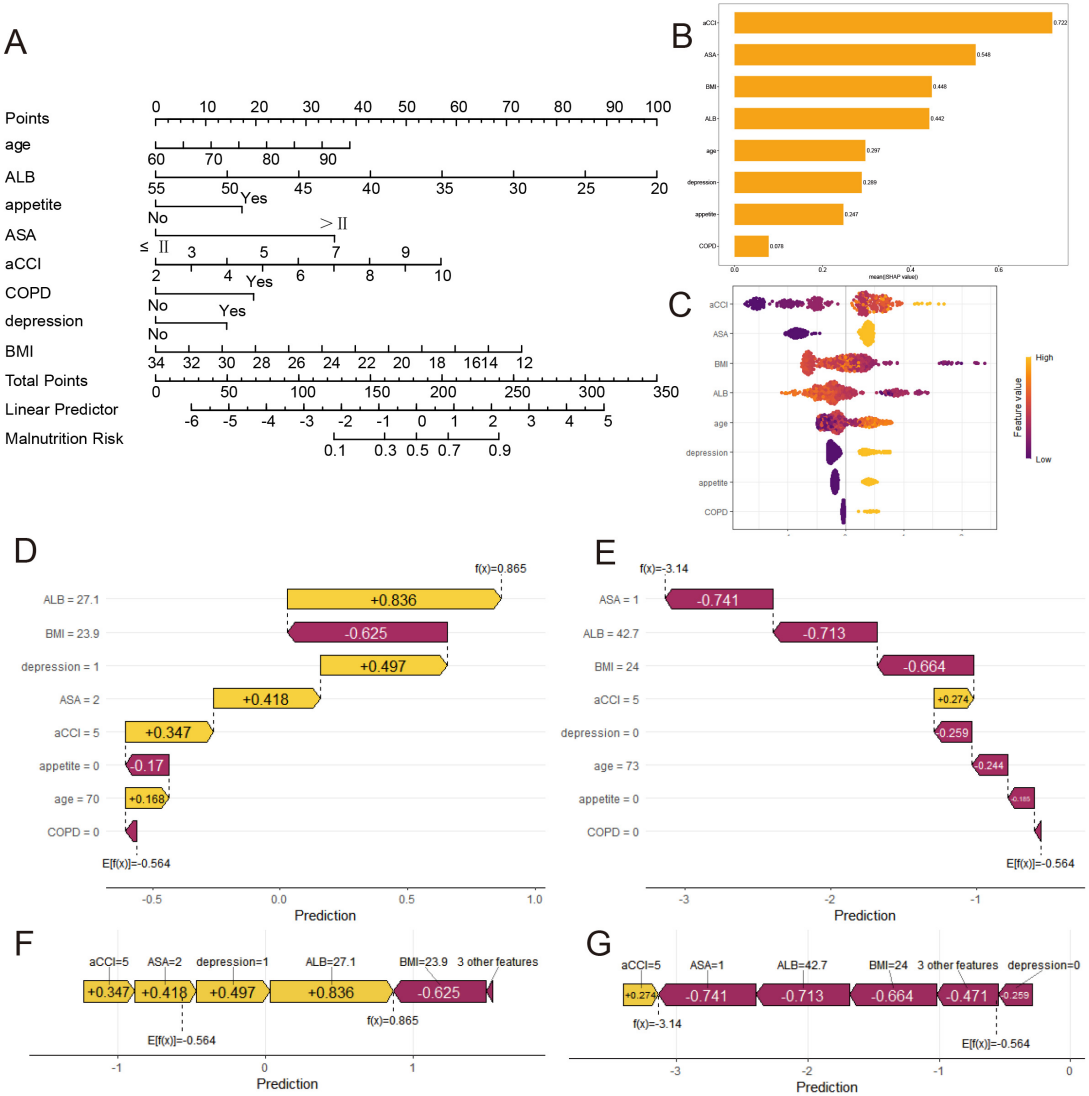
Nomogram and SHAP-based model interpretation. **(A)** Nomogram for predicting malnutrition risk in older adults with hip fractures. **(B)** Bar plot of variable importance ranked by mean absolute SHAP values. **(C)** SHAP beeswarm plot showing the distribution of SHAP values for each feature. **(D)** SHAP waterfall plot for a representative positive case. **(E)** SHAP waterfall plot for a representative negative case. **(F)** SHAP force plot for a positive prediction. **(G)** SHAP force plot for a negative prediction.

The above mentioned results indicate that while the XGBoost model showed strong performance in identifying older hip fracture patients with malnutrition, the LR model demonstrated relatively more stable performance in different clinical settings. Therefore, both models were visually presented to facilitate their clinical translation. For the LR model, a nomogram was developed to estimate the probability of malnutrition in older hip fracture patients, as shown in Figure 5A. For each variable included, a vertical line is drawn upward from the patient's value to intersect the top axis at the corresponding score on the “Points” scale. The sum of these individual scores yields the total points, which represents the patient's overall estimated probability of malnutrition. A vertical line drawn downward from the total points then intersects the bottom axis, indicating the corresponding estimated probability of malnutrition. For example, consider a 70-year-old hip fracture patient with a BMI of 24 kg/m^2^ and an ALB level of 35 g/L. The patient has no documented history of COPD or depression, reports no decreased appetite, has an aCCI score of 3, and a preoperative ASA classification of III or higher.The total points calculated are: 42 + 45 + 58 + 50 + 50 + 50 + 33 + 90 = 418. This total corresponds to a estimated malnutrition probability of 19%. Furthermore, to enhance practicality and streamline the assessment process in clinical settings, we developed and deployed an interactive web-based calculator for malnutrition probability estimation in older hip fracture patients, based on both the LR and XGBoost models. The calculator is accessible at: https://hip-fracture-malnutrition-test.shinyapps.io/dynnomapp/.

This study employed SHAP (Shapley Additive Explanations) values to interpret the outputs of the XGBoost model. At the population level, Figure 5B presents the mean absolute SHAP values for each feature in a bar chart format, representing their overall importance in the model's classifications. The features, ranked from highest to lowest importance, are: aCCI (0.72), ASA classification (0.55), BMI (0.45), ALB (0.44), age (0.30), depression (0.29), decreased appetite (0.25), and COPD (0.08). The highest mean absolute SHAP value for aCCI indicates that it exerts the greatest relative influence on the model's outputs, followed by ASA classification. Figure 5C displays the distribution of SHAP values for each feature. Each point represents an individual sample, colored according to its feature value (purple for low, yellow for high). The sign and magnitude of the SHAP value indicate the direction and degree of the feature's influence on model output. A positive SHAP value increases the estimated probability of malnutrition, while a negative value decreases it. A larger absolute SHAP value indicates a stronger contribution of the feature to the model output. Greater deviation from zero indicates stronger feature contribution. As shown, higher aCCI, advanced age, ASA classification > II, depression, decreased appetite, and COPD were associated with positive SHAP values, indicating increased probability of malnutrition. In contrast, higher BMI and ALB levels correlated with negative SHAP values, indicating their contribution to a lower estimated probability of malnutrition.

Figures 5D, E display the SHAP values and final model output for a representative malnutrition-positive case. Key feature contributions were: ALB 27.1 g/L (+0.84), BMI 23.9 kg/m^2^ (−0.63), depression (+0.50), ASA classification > II (+0.42), aCCI score 5 (+0.35), no decreased appetite (−0.17), age 70 years (+0.17), and no COPD (−0.04). With a baseline value E[f(x)] = −0.56, the sum yielded a final classification score f(x) = 0.87. As this value exceeds 0, the patient was classified as malnourished. Figures 5F, G present the SHAP values and final model output for a nutritionally normal (negative) case. The feature contributions were: ASA classification ≤ II (−0.74), ALB 42.7 g/L (−0.71), BMI 24 kg/m^2^ (−0.66), aCCI score 5 (+0.27), no depression (−0.26), age 73 years (−0.24), no decreased appetite (−0.19), and no COPD (−0.04). Using the same baseline value E[f(x)] = −0.56, the final classification score value f(x) was −3.14. Since this value is less than 0, the patient was classified as non-malnourished.

## 4 Discussion

### 4.1 Malnutrition prevalence and patient characteristics

Using the GLIM criteria, this study assessed the nutritional status of 526 older hip fracture patients and identified 204 cases of malnutrition, yielding a prevalence rate of 38.78 %. This finding aligns with previous research: Li et al. (9) reported malnutrition prevalence rates of 39.01 and 35.19% in two cohorts of 182 and 216 older hip fracture patients, respectively. Similarly, Wu et al. (30) observed a 41.6% prevalence of malnutrition among 161 patients, with 26.7% classified as moderate and 14.9% as severe. These results are broadly consistent with existing evidence in Chinese populations and further support the substantial burden of malnutrition among older hip fracture patients. Collectively, the findings emphasize the urgent need to establish effective and standardized nutritional management strategies and highlight the critical importance of early identification of malnourished individuals to facilitate timely and targeted nutritional interventions.

### 4.2 Risk factors for malnutrition and their importance

The results of this study indicate a significant positive association between aCCI and malnutrition status in older hip fracture patients. This finding is supported by Li et al. (27), who reported that patients with more than three comorbidities had an approximately 9.5-fold higher odds of malnutrition compared with those having three or fewer. An elevated aCCI score reflects a greater burden of chronic comorbidities, which is linked to heightened systemic inflammatory activity. The persistent release of pro-inflammatory cytokines further activates the ubiquitin–proteasome system and suppresses the mTOR signaling pathway, accelerating skeletal muscle protein catabolism and leading to muscle atrophy and a negative nitrogen balance (31). Furthermore, dysfunction of vital organs may impair the biosynthesis of nutritional biomarkers such as albumin, increase energy expenditure, and reduce nutrient absorption efficiency, thereby promoting a catabolic-dominant metabolic state (32). Additionally, multimorbidity is frequently accompanied by polypharmacy, in which certain medications may directly suppress appetite or aggravate electrolyte imbalances, further compromising nutrient intake and metabolic balance (33). Collectively, these mechanisms act synergistically to create a vicious cycle of inflammation, metabolic dysregulation, and functional decline, thereby contributing to the presence and severity of malnutrition in older hip fracture patients.

Patients with ASA classification > II had a 2.91 times higher odds of malnutrition compared to those with ASA ≤ II, consistent with Venianaki et al. (34). A higher ASA grade reflects reduced physiological reserve and systemic functional decline, which heightens neuroendocrine and inflammatory responses to physiological stressors such as trauma or surgery, thereby promoting skeletal muscle catabolism and depletion of nutritional stores (35). Moreover, elevated ASA grades are associated with a higher incidence of postoperative complications (e.g., heart failure, pneumonia) (36) and adverse clinical outcomes (e.g., prolonged hospitalization, need for intensive care, mortality) (37), which collectively impair the body's ability to compensate for energy deficits and contribute to impaired nutritional status.

BMI, a simple and widely used indicator for nutritional assessment, showed an inverse relationship with malnutrition status in this study, consistent with Li et al. (9). A low BMI reflects reduced body energy stores. During acute trauma, hypermetabolism combined with insufficient energy reserves can intensify negative energy balance, enhance gluconeogenesis, and ultimately contribute to malnutrition (35). ALB, a hepatic-derived plasma protein with a relatively long half-life, serves as an indicator of chronic nutritional status and is widely used to monitor hip fracture patients (38). Our results confirmed a significant negative correlation between ALB levels and malnutrition status. During periods of acute inflammatory or hypermetabolic stress, serum ALB acts as a negative acute-phase protein, and its levels decline markedly owing to hemodilution and extravascular redistribution rather than actual protein loss (39). Reduced colloid osmotic pressure caused by hypoalbuminemia may aggravate tissue edema and compromise gastrointestinal absorption efficiency. Furthermore, as a key transport protein, decreased ALB levels may hinder the delivery of nutrients and hormones to target tissues, thereby adversely affecting nutritional status (40).

Age is a well-recognized, non-modifiable factor strongly associated with malnutrition. Physiological decline associated with aging—including reduced digestive and absorptive capacity, and a decline in smell and taste perception—impairs nutrient intake and utilization, thereby contributing to the presence and severity of malnutrition in older adults (41). Consistent with epidemiological trends, the prevalence of multimorbidity exhibits a clear age-dependent increase, rising from 32.4% among those aged 60–69 to 38.5% in the 70–79 group and reaching 40.2% in individuals aged ≥80 years (42). Chronic inflammation and polypharmacy linked to multimorbidity can disrupt metabolic homeostasis and are associated with an increased prevalence of malnutrition. In this study, decreased appetite—assessed by patient self-report—was associated with a higher likelihood of malnutrition compared with patients with normal appetite, consistent with Fielding et al. (43). As a key component of the MNA-SF, there is a bidirectional relationship between decreased appetite and malnutrition. On one hand, appetite loss directly reduces energy intake, leading to progressive weight loss, sarcopenia, and frailty, thereby diminishing the body's ability to meet hypermetabolic demands under inflammatory stress. Conversely, deficiencies in essential micronutrients can contribute to appetite loss, which may be further aggravated by underlying diseases and medication effects, thus creating a self-perpetuating cycle of impaired nutritional status (43).

The results of this study indicate that depression and COPD were associated with an increased odds of malnutrition (depression: OR = 1.72; COPD: OR = 2.11), which is broadly consistent with the findings reported by He Zhiqiang et al. (21). Previous studies (44) have shown that depression-related a motivation and anhedonia can markedly reduce voluntary food intake, leading to decreased nutrient absorption efficiency. Moreover, depressive symptoms such as low mood and reduced physical activity often result in neglect of personal health and a passive attitude toward recovery, further contributing to the imbalance between dietary intake and metabolic consumption. The pathogenesis of COPD-related malnutrition mainly involves chronic systemic inflammation and disorders of energy metabolism. In addition, increased airway resistance and mucus hypersecretion cause a functional competition between breathing and eating, forcing patients to reduce food intake to alleviate dyspnea, thereby compromising overall nutritional intake (45).

Feature importance analysis revealed that aCCI emerged as the most influential feature across both the RF and XGBoost models. The relative importance of other variables, however, varied between the two due to differences in algorithmic mechanisms and methods of importance quantification. For instance, decreased appetite ranked second in the RF model but notably lower (around sixth) in XGBoost, possibly reflecting interaction effects between decreased appetite and aCCI. The RF algorithm, which ensembles multiple decision trees through bagging, is particularly effective in capturing such feature interactions. In contrast, XGBoost prioritizes features primarily according to their direct gain in splitting purity; thus, the independent contribution of decreased appetite—partially captured by aCCI—may be downweighted, resulting in its lower ranking.

Furthermore, the importance ranking of the same feature within a single model can differ depending on the evaluation metric. For example, in the XGBoost model, ALB ranked second based on Gain but fell to fourth when evaluated by SHAP values. This inconsistency may arise because ALB, though frequently selected for high-impact splits, exhibits heterogeneous effects on malnutrition status across distinct patient subgroups. Such bidirectional effects reduce its mean |SHAP value|, as visually confirmed in the beeswarm plot (Figure 5C). Moreover, variations in feature distributions between the training and validation datasets may also account for discrepancies in importance ranking. Therefore, caution should be exercised when interpreting feature importance outcomes.

### 4.3 Development and evaluation of the diagnostic model

This study optimized the hyperparameter settings of LR, RF, SVM, XGBoost, and ANN algorithms, thereby constructing five diagnostic models for malnutrition among older patients with hip fracture. Model performance evaluation showed that both XGBoost and RF achieved higher AUC values and better confusion matrix–based metrics than the other algorithms in the training dataset. Although the difference in AUC between these two top-performing models was not statistically significant (ΔAUC = 0.0059), even marginal improvements may be clinically meaningful given the serious complications and substantial healthcare costs associated with hip fracture (46). After a comprehensive assessment of calibration performance and clinical applicability, the XGBoost model was ultimately selected as the optimal diagnostic model—a conclusion further validated by its robust internal validation results. Previous studies (47) have also confirmed the advantages of XGBoost in structured data classification and regression, owing to its flexibility, ability to model complex nonlinear relationships, inherent regularization, and computational efficiency for large-scale datasets. The LR model yielded the lowest AUC (0.89), closely aligning with the 0.89 reported by Li et al. (9). To our knowledge, no prior research has developed malnutrition diagnostic prediction models for older hip fracture patients based on other machine learning algorithms, rendering direct performance comparisons unavailable and underscoring the novelty of this study.

Compared with the training set, all models showed a decline in performance metrics on the external validation set. This decline may be partially attributable to distributional shifts between the training and validation datasets. Significant differences observed in variables such as education level, fracture type, laboratory parameters (e.g., creatinine, AST), and perioperative factors may have affected the calibration performance of the models, as variations in feature distributions can alter the relationship between predictors and outcome probabilities. This decrease is likely attributable to distributional shifts between the training and validation datasets. Although the validation cohort was derived from an independent institution, all participating hospitals were located within the same city, which may limit population heterogeneity. Nevertheless, statistically significant differences in several clinical and perioperative variables were observed, which may have altered the underlying predictor–outcome relationships and contributed to the observed decline in calibration performance. These findings highlight the sensitivity of machine learning models to dataset shifts, even within geographically proximate populations. Univariate analysis revealed significant differences in several variables, including education level, fracture type, Cr, AST, operation duration, anesthesia duration, postoperative drainage volume, and postoperative bed-rest duration. These variations could be explained by differences in institutional expertise, treatment and rehabilitation protocols, laboratory standards, and regional patient characteristics. Moreover, model performance may have been affected by methodological limitations such as sampling bias and data collection accuracy. Although some degradation was anticipated, all evaluation metrics remained within clinically acceptable ranges. Nonetheless, this finding reflects the fragmentation of healthcare data systems in China, highlighting the need for better integration of heterogeneous clinical information across institutions. Notably, RF and XGBoost demonstrated a more pronounced drop in performance than other algorithms, with statistically significant differences across datasets. This higher sensitivity likely stems from their greater vulnerability to data distribution shifts. Complex algorithms such as RF and XGBoost are particularly prone to performance decline caused by biased training data or limited sample sizes (48). Despite this, both models still achieved acceptable classification accuracy and discrimination in the external validation set, without statistically significant AUC differences compared to other models. Therefore, it would be premature to question their applicability for identifying malnutrition in older hip fracture patients. Further research involving larger and more diverse validation cohorts is warranted to confirm their robustness and generalizability.

DCA demonstrated satisfactory clinical utility for all models across the training, internal, and external validation sets, with net benefits exceeding the “treat-all” and “treat-none” strategies across a range of threshold probabilities. The XGBoost and RF models showed superior performance, with the widest applicable threshold ranges. Dynamically calibrating intervention thresholds remains a central challenge in clinical translation, requiring careful balance between diagnostic sensitivity and the efficient allocation of healthcare resources. Although this study did not establish definitive intervention thresholds, previously published methods for threshold determination provide a valuable reference framework for defining clinical decision points. Parsons et al. (49) emphasized that incorporating health economic factors (e.g., net monetary benefit) into the selection of intervention thresholds can transform clinical decision support systems into value-based healthcare models, enabling better trade-offs between the consequences of correct and incorrect classifications. Similarly, Cui et al. (50) determined an intervention threshold for the FRAX^®^ model in a Chinese postmenopausal osteoporosis population through health economic evaluation. Their Markov model simulations using real-world data suggested that setting the 10-year major osteoporotic fracture risk threshold at 7% would optimize cost-effective anti-osteoporosis treatment. These studies indicate that quantifying clinical net benefit across probability thresholds, combined with cost-effectiveness analyses, can effectively guide the selection of optimal intervention thresholds while maximizing cost-efficiency. Overall, all models achieved acceptable discriminative performance and satisfactory generalizability during training, internal, and external validation, highlighting their potential for clinical implementation.

This study has several limitations. First, although external validation was performed, the validation dataset was derived from a single center within the same geographic region as the training cohort, which may limit the generalizability of the model to populations with different demographic characteristics and healthcare settings. In addition, differences observed in several variables between the training and validation sets suggest the presence of distributional shifts, which may have altered predictor–outcome relationships and contributed to the decline in calibration performance in the external validation. Second, no model recalibration was applied to the external validation dataset in the present study. Future research may consider recalibration strategies, such as intercept adjustment or Platt scaling, to improve model calibration across diverse clinical settings. Furthermore, as body mass index (BMI) and decreased appetite are components of the GLIM criteria, the model may be subject to potential incorporation bias. However, sensitivity analysis excluding these variables demonstrated that the model retained good discriminative performance (AUC > 0.80), suggesting that its predictive ability is not solely dependent on the diagnostic criteria themselves. In addition, given the conceptual overlap between the age-adjusted Charlson Comorbidity Index (aCCI) and the etiologic domain of GLIM, the model should be interpreted as a diagnostic screening aid rather than an independent diagnostic standard. Third, the relatively small external validation sample size, along with differences in feature distributions between datasets, may affect model stability. Moreover, the study population was limited to cognitively intact patients undergoing surgical treatment, which may restrict the applicability of the findings to broader populations. Finally, the lack of standardized protocols for multicenter data collection may affect data consistency and limit the transferability of the model across institutions.

## 5 Conclusions

In summary, this study revealed a high prevalence of malnutrition among older hip fracture patients. Through multi-stage feature selection, eight core variables were ultimately incorporated into the models: age, BMI, decreased appetite, COPD, aCCI, depression, ALB, and ASA classification, with aCCI recognized as the most important feature. Although aCCI overlaps with the GLIM etiologic domain, it does not duplicate any diagnostic component. It provides a composite measure of comorbidity burden beyond the GLIM framework. During model training and validation, the XGBoost model demonstrated superior classification accuracy, whereas the LR model demonstrated satisfactory stability and generalizability. Both models were deployed as web-based visualization tools to provide an efficient, accurate, and accessible means for identifying malnutrition in older hip fracture patients. Future research should include large-scale, multi-center studies across diverse regions to further validate model generalizability and transferability. Furthermore, the classification results generated by these models could guide the development of systematic and individualized perioperative nutritional care protocols, thereby advancing nursing practice from experience-driven to data-driven paradigms.

## Data Availability

The original contributions presented in the study are included in the article/Supplementary material, further inquiries can be directed to the corresponding author.
