# Supplementary material for: Development and validation of a machine learning–based model for diagnosing perioperative malnutrition in older adults with hip fracture
Source: Front Nutr. 2026 Jun 8;13:1751993. doi: 10.3389/fnut.2026.1751993 (PMC13284978; doi:10.3389/fnut.2026.1751993)
Supplement: Supplementary file 1 [file Table_1.docx]

**Table S1.** GLIM Consensus on Malnutrition Diagnosis

| **Item / Indicator** | **Criteria / Cut-off** |
| --- | --- |
| Step 1: Screening |  |
| MNA-SF | ≤11 within 24 hours of admission |
| Step 2: Phenotypic criteria |  |
| Weight loss | >5% within 6 months or >10% beyond 6 months |
| Low BMI | <18.5 kg/m² (aged <70 years); <20 kg/m² (aged ≥70 years) |
| CC | Male <34 cm; female <33 cm |
| HGS | Male <28 kg; female <18 kg |
| MUAC | Male <22 cm; female <21 cm |
| TSF | Male <0.7 cm; female <1.2 cm |
| MAMC | Male <22.3 cm; female <18.6 cm |
| Step 2: Etiologic criteria |  |
| Food intake / absorption | ≤50% energy requirements for 1 week, or reduced intake >2 weeks, or chronic gastrointestinal disease |
| Disease burden / inflammation | Acute illness/trauma (e.g., hip fracture) or chronic disease-related inflammation |
| Step 3: Malnutrition diagnosis |  |
| Diagnostic criteria | At least one phenotypic criterion + one etiologic criterion |
| Step 4: Severity grading |  |
| Moderate malnutrition | Weight loss 5%-10% (6 months) or 10%-20% (>6 months); or BMI below age-specific cut-off |
| Severe malnutrition | Weight loss >10% (6 months) or >20% (>6 months); or BMI <17.0 kg/m² (<70 years) or <17.8 kg/m² (≥70 years) |

**Table S2.** Missing data patterns of candidate variables

| **Variable** | **Total (N=526)** | | **Training set (N=385)** | | **External validation set (N=141)** | |
| --- | --- | --- | --- | --- | --- | --- |
|  | **Missing frequency** | **Missing proportion** | **Missing frequency** | **Missing proportion** | **Missing frequency** | **Missing proportion** |
| CRP | 77 | 14.64% | 36 | 9.35% | 41 | 29.08% |
| 25-(OH)VD3 | 290 | 55.13% | 169 | 43.90% | 121 | 85.82% |
| TC | 188 | 35.74% | 145 | 37.66% | 43 | 30.50% |
| TG | 197 | 37.45% | 154 | 40.00% | 43 | 30.50% |
| HDL | 197 | 37.45% | 154 | 40.00% | 43 | 30.50% |
| LDL | 197 | 37.45% | 154 | 40.00% | 43 | 30.50% |

**Table S3.** Comparison of Disease-Related Factors Between the Training and Validation Sets

| **Variables** | **Overall（N=526）** | **Training Set（N=385）** | **External Validation Set（N=141）** | **Statistics** | **P value** |
| --- | --- | --- | --- | --- | --- |
| Myocardial Infarction / Coronary Artery Disease |  |  |  | χ^2^=1.36 | 0.24 |
| No | 410 (77.95) | 305 (79.22) | 105 (74.47) |  |  |
| Yes | 116 (22.05) | 80 (20.78) | 36 (25.53) |  |  |
| Congestive Heart Failure |  |  |  | χ^2^=0.004 | 0.95 |
| No | 493 (93.73) | 361 (93.77) | 132 (93.62) |  |  |
| Yes | 33 (6.27) | 24 (6.23) | 9 (6.38) |  |  |
| Peripheral Vascular Diseases |  |  |  | χ^2^=0.21 | 0.65 |
| No | 365 (69.39) | 265 (68.83) | 100 (70.92) |  |  |
| Yes | 161 (30.61) | 120 (31.17) | 41 (29.08) |  |  |
| Cerebrovascular Disorders or Transient Ischemic Attack |  |  |  | χ^2^=2.41 | 0.12 |
| No | 349 (66.35) | 248 (64.42) | 101 (71.63) |  |  |
| Yes | 177 (33.65) | 137 (35.58) | 40 (28.37) |  |  |
| Hemiplegia |  |  |  | - | 0.45 |
| No | 518 (98.48) | 380 (98.70) | 138 (97.87) |  |  |
| Yes | 8 (1.52) | 5 (1.30) | 3 (2.13) |  |  |
| Dementia / Alzheimer Disease |  |  |  | χ^2^=0.30 | 0.58 |
| No | 483 (91.83) | 352 (91.43) | 131 (92.91) |  |  |
| Yes | 43 (8.17) | 33 (8.57) | 10 (7.09) |  |  |
| COPD |  |  |  | χ^2^=0.94 | 0.33 |
| No | 477 (90.68) | 352 (91.43) | 125 (88.65) |  |  |
| Yes | 49 (9.32) | 33 (8.57) | 16 (11.35) |  |  |
| Rheumatoid Arthritis / Connective Tissue Diseases |  |  |  | - | 0.38 |
| No | 511 (97.15) | 372 (96.62) | 139 (98.58) |  |  |
| Yes | 15 (2.85) | 13 (3.38) | 2 (1.42) |  |  |
| Neoplasms |  |  |  | χ^2^=0.16 | 0.69 |
| No | 503 (95.63) | 369 (95.84) | 134 (95.04) |  |  |
| Yes | 23 (4.37) | 16 (4.16) | 7 (4.96) |  |  |
| Diabetes Mellitus |  |  |  | χ^2^=0.48 | 0.49 |
| No | 381 (72.43) | 282 (73.25) | 99 (70.21) |  |  |
| Yes | 145 (27.57) | 103 (26.75) | 42 (29.79) |  |  |
| Moderate to Severe Chronic Kidney Disease |  |  |  | χ^2^=2.72 | 0.10 |
| No | 477 (90.68) | 354 (91.95) | 123 (87.23) |  |  |
| Yes | 49 (9.32) | 31 (8.05) | 18 (12.77) |  |  |
| Peptic Ulcer |  |  |  | - | 0.61 |
| No | 521 (99.05) | 382 (99.22) | 139 (98.58) |  |  |
| Yes | 5 (0.95) | 3 (0.78) | 2 (1.42) |  |  |
| Liver Diseases |  |  |  | χ^2^=2.84 | 0.09 |
| No | 493 (93.73) | 365 (94.81) | 128 (90.78) |  |  |
| Yes | 33 (6.27) | 20 (5.19) | 13 (9.22) |  |  |
| Hypertension |  |  |  | χ^2^=0.56 | 0.45 |
| No | 221 (42.02) | 158 (41.04) | 63 (44.68) |  |  |
| Yes | 305 (57.98) | 227 (58.96) | 78 (55.32) |  |  |
| Vision Disorders |  |  |  | - | 1.00 |
| No | 518 (98.48) | 379 (98.44) | 139 (98.58) |  |  |
| Yes | 8 (1.52) | 6 (1.56) | 2 (1.42) |  |  |
| Chronic Gastritis |  |  |  | - | 0.71 |
| No | 517 (98.29) | 379 (98.44) | 138 (97.87) |  |  |
| Yes | 9 (1.71) | 6 (1.56) | 3 (2.13) |  |  |
| aCCI | 5 (3, 6) | 5 (3, 6) | 5 (3, 7) | Z=﹣0.60 | 0.55 |
| Sleep Wake Disorders |  |  |  | χ^2^=2.16 | 0.14 |
| No | 340 (64.64) | 256 (66.49) | 84 (59.57) |  |  |
| Yes | 186 (35.36) | 129 (33.51) | 57 (40.43) |  |  |
| Depression |  |  |  | χ^2^=0.02 | 0.88 |
| No | 378 (71.86) | 276 (71.69) | 102 (72.34) |  |  |
| Yes | 148 (28.14) | 109 (28.31) | 39 (27.66) |  |  |
| Oral Health |  |  |  | χ^2^=1.11 | 0.29 |
| Good | 318 (60.46) | 238 (61.82) | 80 (56.74) |  |  |
| Poor | 208 (39.54) | 147 (38.18) | 61 (43.26) |  |  |
| Swallowing Function |  |  |  | χ^2^=0.01 | 0.91 |
| Normal | 480 (91.25) | 351 (91.17) | 129 (91.49) |  |  |
| Abnormal | 46 (8.75) | 34 (8.83) | 12 (8.51) |  |  |
| Type of Hip Fracture |  |  |  | χ^2^=7.43 | **0.01** |
| Femoral Neck Fracture | 375 (71.29) | 287 (74.55) | 88 (62.41) |  |  |
| Intertrochanteric/Subtrochanteric Fracture | 151 (28.71) | 98 (25.45) | 53 (37.59) |  |  |
| Time from Fracture to Surgery (h) | 155 (109, 258) | 153 (107, 253) | 166 (114, 274) | Z=﹣1.83 | 0.07 |

**Note.** Mean +/- SD; Median (P25, P75); n (%); chi-square test (χ^2^); Mann-Whitney U test (Z); Fisher's exact test (-); Transient Ischemic Attack (TIA); Chronic Obstructive Pulmonary Disease (COPD); Age-adjusted Charlson Comorbidity Index (aCCI).

**Table S4.** Comparison of Blood Biochemical Indices Between the Training and Validation Sets

| **Variables** | **Overall（N=526）** | **Training Set（N=385）** | **External Validation Set（N=141）** | **Statistics** | **P value** |
| --- | --- | --- | --- | --- | --- |
| HGB（g/L） | 118.59±20.33 | 119.30±19.30 | 116.65±22.85 | t=1.22 | 0.22 |
| WBC（10^9^/L） | 7.34 (5.94, 8.91) | 7.44 (6.00, 8.84) | 7.06 (5.72, 8.94) | Z=﹣0.85 | 0.40 |
| LYM（10^9^/L） | 1.08 (0.79, 1.40) | 1.05 (0.78, 1.34) | 1.14 (0.83, 1.54) | Z=﹣1.91 | 0.06 |
| NEUT（10^9^/L） | 5.50 (4.11, 7.10) | 5.57 (4.21, 7.10) | 5.23 (3.81, 6.99) | Z=﹣1.15 | 0.25 |
| PLT（10^9^/L） | 187 (152, 229) | 191 (153, 234) | 181 (148, 215) | Z=﹣1.43 | 0.15 |
| NLR | 5.05 (3.19, 8.18) | 5.11 (3.39, 8.29) | 4.57 (2.78, 8.02) | Z=﹣1.76 | 0.08 |
| PLR | 168.93 (129.89, 241.23) | 175.68 (134.19, 246.59) | 155.56 (117.82, 224.84) | Z=﹣2.46 | **0.01** |
| CRP（mg/L） | 21.40 (8.64, 40.18) | 19.33 (8.56, 40.30) | 24.79 (10.53, 38.97) | Z=﹣0.46 | 0.64 |
| TP（g/L） | 63.30±7.20 | 63.52±7.10 | 62.68±7.46 | t=1.18 | 0.24 |
| ALB（g/L） | 37.34±4.99 | 37.37±4.91 | 37.27±5.21 | t=0.21 | 0.84 |
| PA（mg/L） | 167.60 (130.00, 205.90) | 163.00 (130.00, 197.00) | 187.60 (135.50, 224.00) | Z=﹣3.32 | **＜0.001** |
| GLU（mmol/L） | 5.63 (4.93, 6.75) | 5.62 (4.94, 6.62) | 5.71 (4.89, 6.80) | Z=﹣0.38 | 0.71 |
| Ca^2+^（mmol/L） | 2.16 (2.06, 2.24) | 2.15 (2.05, 2.24) | 2.19 (2.08, 2.25) | Z=﹣2.41 | **0.02** |
| Cr（umol/L） | 56.00 (46.00, 70.00) | 57.00 (47.00, 70.00) | 52.45 (42.70, 70.30) | Z=﹣2.02 | **0.04** |
| BUN（mmol/L） | 6.00 (4.80, 7.70) | 6.00 (4.80, 7.70) | 6.00 (4.80, 8.17) | Z=﹣0.45 | 0.65 |
| ALT（U/L） | 15.00 (11.00, 21.00) | 15.00 (11.50, 20.10) | 14.00 (11.00, 22.00) | Z=﹣0.13 | 0.90 |
| AST（U/L） | 20.25 (17.00, 25.95) | 20.00 (17.00, 25.00) | 22.00 (18.00, 26.00) | Z=﹣2.84 | **0.01** |

**Note.** Mean +/- SD; Median (P25, P75); n (%); Student’s t-test (t); chi-square test (χ^2^); Mann-Whitney U test (Z); Haemoglobin (HGB); White Blood Cell Count (WBC); Lymphocyte Count (LYM); Neutrophil Count (NEUT); Platelet Count (PLT); Neutrophil-to-Lymphocyte Ratio (NLR); Platelet-to-Lymphocyte Ratio (PLR); C-Reactive Protein (CRP); Total Protein (TP); Albumin (ALB); Prealbumin (PA); Glucose (GLU); Calcium (Ca²⁺); Creatinine (Cr); Blood Urea Nitrogen (BUN); Alanine Aminotransferase (ALT); Aspartate Aminotransferase (AST).

**Table S5.** Comparison of Surgery-Related Factors Between the Training and Validation Sets

| **Variables** | **Overall（N=526）** | **Training Set（N=385）** | **External Validation Set（N=141）** | **Statistics** | **P value** |
| --- | --- | --- | --- | --- | --- |
| Type of Surgery |  |  |  | χ^2^=2.73 | 0.26 |
| Total Hip Arthroplasty | 303 (57.60) | 229 (59.48) | 74 (52.48) |  |  |
| Partial Hip Arthroplasty | 75 (14.26) | 55 (14.29) | 20 (14.18) |  |  |
| Open Reduction and Internal Fixation | 148 (28.14) | 101 (26.23) | 47 (33.33) |  |  |
| Type of Anesthesia |  |  |  | χ^2^=0.77 | 0.38 |
| General Anesthesia | 439 (83.46) | 318 (82.60) | 121 (85.82) |  |  |
| Neuraxial Anesthesia | 87 (16.54) | 67 (17.40) | 20 (14.18) |  |  |
| Duration of Surgery (min) | 105 (81.25, 130) | 100 (80, 125) | 120 (95, 145) | Z=﹣4.42 | **＜0.001** |
| Duration of Anesthesia (min) | 135 (110, 160) | 130 (105, 155) | 145 (120, 170) | Z=﹣4.51 | **＜0.001** |
| Intraoperative Blood Loss (mL) | 200 (100, 300) | 200 (100, 300) | 150 (100, 200) | Z=﹣1.30 | 0.19 |
| Intraoperative Blood Transfusion |  |  |  | χ^2^=0.14 | 0.71 |
| No | 372 (70.72) | 274 (71.17) | 98 (69.50) |  |  |
| Yes | 154 (29.28) | 111 (28.83) | 43 (30.50) |  |  |
| ASA Physical Status Classification |  |  |  | χ^2^=0.002 | 0.97 |
| ≤ II | 191 (36.31) | 140 (36.36) | 51 (36.17) |  |  |
| II | 335 (63.69) | 245 (63.64) | 90 (63.83) |  |  |
| Preoperative Fasting Time for Clear Fluids (h) | 6 (6, 8) | 6 (6, 8) | 6 (6, 8) | Z=﹣1.71 | 0.09 |
| Preoperative Fasting Time for Solids (h) | 8 (8, 10) | 8 (8, 10) | 8 (8, 8) | Z=﹣0.76 | 0.45 |
| Postoperative Drainage Volume (mL) | 80 (45, 125) | 75 (40, 125) | 90 (55, 140) | Z=﹣2.19 | **0.03** |
| Postoperative Bed Rest Duration (d) | 5 (4, 7) | 5 (4, 7) | 6 (4, 8) | Z=﹣2.82 | **0.01** |

**Note.** Median (P25, P75); n (%); chi-square test (χ^2^); Mann-Whitney U test (Z); American Society of Anesthesiologists (ASA).

**Table S6.** Comparison of Medication Use and Diet-Related Factors Between the Training and Validation Sets

| **Variables** | **Overall（N=526）** | **Training Set（N=385）** | **External Validation Set（N=141）** | **Statistics** | **P value** |
| --- | --- | --- | --- | --- | --- |
| Antihypertensive Drugs |  |  |  | χ^2^=0.26 | 0.61 |
| No | 218 (41.44) | 157 (40.78) | 61 (43.26) |  |  |
| Yes | 308 (58.56) | 228 (59.22) | 80 (56.74) |  |  |
| Lipid-Lowering Drugs |  |  |  | χ^2^=1.02 | 0.31 |
| No | 486 (92.40) | 353 (91.69) | 133 (94.33) |  |  |
| Yes | 40 (7.60) | 32 (8.31) | 8 (5.67) |  |  |
| Hypoglycemic Drugs |  |  |  | χ^2^=0.10 | 0.75 |
| No | 371 (70.53) | 273 (70.91) | 98 (69.50) |  |  |
| Yes | 155 (29.47) | 112 (29.09) | 43 (30.50) |  |  |
| Diuretics |  |  |  | χ^2^=4.11 | **0.04** |
| No | 452 (85.93) | 338 (87.79) | 114 (80.85) |  |  |
| Yes | 74 (14.07) | 47 (12.21) | 27 (19.15) |  |  |
| Hormone Drugs |  |  |  | χ^2^=0.28 | 0.60 |
| No | 497 (94.49) | 365 (94.81) | 132 (93.62) |  |  |
| Yes | 29 (5.51) | 20 (5.19) | 9 (6.38) |  |  |
| Acid-Suppressing |  |  |  | χ^2^=5.55 | **0.02** |
| No | 261 (49.62) | 203 (52.73) | 58 (41.13) |  |  |
| Yes | 265 (50.38) | 182 (47.27) | 83 (58.87) |  |  |
| Antipsychotic Drugs |  |  |  | χ^2^=0.42 | 0.52 |
| No | 466 (88.59) | 339 (88.05) | 127 (90.07) |  |  |
| Yes | 60 (11.41) | 46 (11.95) | 14 (9.93) |  |  |
| Nutritional Supplements |  |  |  | χ^2^=1.68 | 0.20 |
| No | 450 (85.55) | 334 (86.75) | 116 (82.27) |  |  |
| Yes | 76 (14.45) | 51 (13.25) | 25 (17.73) |  |  |
| Daily Protein Intake (g) | 38.90 (34.20, 44.27) | 39.00 (34.30, 44.30) | 38.80 (33.80, 44.20) | Z=﹣0.52 | 0.60 |
| Daily Carbohydrate Intake (g) | 138.15 (130.70, 143.88) | 137.20 (130.80, 143.20) | 138.60 (129.40, 144.50) | Z=﹣0.56 | 0.58 |
| Daily Dietary Fiber Intake (g) | 26.40 (22.13, 29.50) | 26.20 (21.50, 29.40) | 28.10 (23.20, 31.20) | Z=﹣1.98 | **0.05** |
| Daily Fat Intake (g) | 26.6 (22.8, 30.6) | 26.6 (22.6, 30.4) | 26.9 (23.4, 31.9) | Z=﹣1.42 | 0.16 |
| Daily Energy Intake (kcal) | 1371.5 (1282.0, 1438.0) | 1369.0 (1284.0, 1438.0) | 1374.0 (1270.0, 1438.0) | Z=﹣0.26 | 0.79 |

**Note.** Median (P25, P75); n (%); chi-square test (χ^2^); Mann-Whitney U test (Z).

**Table S7.** Comparison of Nutritional Assessment Indicators Between the Training and Validation Sets

| **Variables** | **Overall（N=526）** | **Training Set（N=385）** | **External Validation Set（N=141）** | **Statistics** | **P value** |
| --- | --- | --- | --- | --- | --- |
| MNA-SF | 10 (8, 12) | 10 (8, 12) | 10 (7, 12) | Z=﹣0.05 | 0.96 |
| HGS（kg） | 16.40 (12.20, 22.08) | 16.20 (12.20, 21.50) | 17.70 (11.60, 22.80) | Z=﹣0.85 | 0.40 |
| CC（cm） | 34.20 (32.12, 36.20) | 34.10 (31.80, 36.20) | 34.30 (32.70, 36.40) | Z=﹣0.67 | 0.50 |
| MUAC（cm） | 25.45 (24.30, 26.80) | 25.50 (24.30, 26.70) | 25.40 (23.60, 26.80) | Z=﹣0.59 | 0.56 |
| TSF（cm） | 1.45 (1.13, 1.82) | 1.45 (1.14, 1.81) | 1.44 (1.12, 1.86) | Z=﹣0.53 | 0.59 |
| MAMC（cm） | 20.56 (19.62, 21.59) | 20.64 (19.68, 21.59) | 20.35 (19.43, 21.59) | Z=﹣1.83 | 0.07 |
| Nutritional Status |  |  |  | χ^2^=0.07 | 0.79 |
| Well-Nourished | 322 (61.22) | 237 (61.56) | 85 (60.28) |  |  |
| Malnourished | 204 (38.78) | 148 (38.44) | 56 (39.72) |  |  |

**Note.** Median (P25, P75); n (%); chi-square test(χ^2^); Mann-Whitney U test (Z). Mini Nutritional Assessment-short Form (MNA-SF); Handgrip Strength (HGS,); Calf Circumference (CC); Mid-upper Arm Circumference (MUAC); Triceps Skinfold (TSF); Mid-arm Muscle Circumference (MAMC).

**Table S8.** Univariate Analysis of Disease-Related Factors for Malnutrition in the Training Set

| **Variables** | **Training Set（N=385）** | **Well-Nourished（N=237）** | **Malnourished（N=148）** | **Statistics** | **P value** |
| --- | --- | --- | --- | --- | --- |
| Myocardial Infarction / Coronary Artery Disease |  |  |  | χ^2^=11.70 | ＜0.001 |
| No | 305 (79.22) | 201 (84.81) | 104 (70.27) |  |  |
| Yes | 80 (20.78) | 36 (15.19) | 44 (29.73) |  |  |
| Congestive Heart Failure |  |  |  | χ^2^=17.94 | ＜0.001 |
| No | 361 (93.77) | 232 (97.89) | 129 (87.16) |  |  |
| Yes | 24 (6.23) | 5 (2.11) | 19 (12.84) |  |  |
| Peripheral Vascular Diseases |  |  |  | χ^2^=16.34 | ＜0.001 |
| No | 265 (68.83) | 181 (76.37) | 84 (56.76) |  |  |
| Yes | 120 (31.17) | 56 (23.63) | 64 (43.24) |  |  |
| Cerebrovascular Disorders or Transient Ischemic Attack |  |  |  | χ^2^=26.08 | ＜0.001 |
| No | 248 (64.42) | 176 (74.26) | 72 (48.65) |  |  |
| Yes | 137 (35.58) | 61 (25.74) | 76 (51.35) |  |  |
| Hemiplegia |  |  |  | - | 0.07 |
| No | 380 (98.70) | 236 (99.58) | 144 (97.30) |  |  |
| Yes | 5 (1.30) | 1 (0.42) | 4 (2.70) |  |  |
| Dementia / Alzheimer Disease |  |  |  | χ^2^=14.90 | ＜0.001 |
| No | 352 (91.43) | 227 (95.78) | 125 (84.46) |  |  |
| Yes | 33 (8.57) | 10 (4.22) | 23 (15.54) |  |  |
| COPD |  |  |  | χ^2^=28.70 | ＜0.001 |
| No | 352 (91.43) | 231 (97.47) | 121 (81.76) |  |  |
| Yes | 33 (8.57) | 6 (2.53) | 27 (18.24) |  |  |
| Rheumatoid Arthritis / Connective Tissue Diseases |  |  |  | - | 1.00 |
| No | 372 (96.62) | 229 (96.62) | 143 (96.62) |  |  |
| Yes | 13 (3.38) | 8 (3.38) | 5 (3.38) |  |  |
| Neoplasms |  |  |  | χ^2^=4.08 | 0.04 |
| No | 369 (95.84) | 231 (97.47) | 138 (93.24) |  |  |
| Yes | 16 (4.16) | 6 (2.53) | 10 (6.76) |  |  |
| Diabetes Mellitus |  |  |  | χ^2^=2.30 | 0.13 |
| No | 282 (73.25) | 180 (75.95) | 102 (68.92) |  |  |
| Yes | 103 (26.75) | 57 (24.05) | 46 (31.08) |  |  |
| Moderate to Severe Chronic Kidney Disease |  |  |  | χ^2^=12.23 | ＜0.001 |
| No | 354 (91.95) | 227 (95.78) | 127 (85.81) |  |  |
| Yes | 31 (8.05) | 10 (4.22) | 21 (14.19) |  |  |
| Peptic Ulcer |  |  |  | - | 0.56 |
| No | 382 (99.22) | 236 (99.58) | 146 (98.65) |  |  |
| Yes | 3 (0.78) | 1 (0.42) | 2 (1.35) |  |  |
| Liver Diseases |  |  |  | χ^2^=0.02 | 0.88 |
| No | 365 (94.81) | 225 (94.94) | 140 (94.59) |  |  |
| Yes | 20 (5.19) | 12 (5.06) | 8 (5.41) |  |  |
| Hypertension |  |  |  | χ^2^=4.30 | 0.040 |
| No | 158 (41.04) | 107 (45.15) | 51 (34.46) |  |  |
| Yes | 227 (58.96) | 130 (54.85) | 97 (65.54) |  |  |
| Vision Disorders |  |  |  | - | 0.03 |
| No | 379 (98.44) | 236 (99.58) | 143 (96.62) |  |  |
| Yes | 6 (1.56) | 1 (0.42) | 5 (3.38) |  |  |
| Chronic Gastritis |  |  |  | - | 0.68 |
| No | 379 (98.44) | 234 (98.73) | 145 (97.97) |  |  |
| Yes | 6 (1.56) | 3 (1.27) | 3 (2.03) |  |  |
| aCCI | 5 (3, 6) | 4 (2, 5) | 6 (5, 7) | Z=﹣10.86 | ＜0.001 |
| Sleep Wake Disorders |  |  |  | χ^2^=68.95 | ＜0.001 |
| No | 256 (66.49) | 195 (82.28) | 61 (41.22) |  |  |
| Yes | 129 (33.51) | 42 (17.72) | 87 (58.78) |  |  |
| Depression |  |  |  | χ^2^=52.30 | ＜0.001 |
| No | 276 (71.69) | 201 (84.81) | 75 (50.68) |  |  |
| Yes | 109 (28.31) | 36 (15.19) | 73 (49.32) |  |  |
| Oral Health |  |  |  | χ^2^=46.12 | ＜0.001 |
| Good | 238 (61.82) | 178 (75.11) | 60 (40.54) |  |  |
| Poor | 147 (38.18) | 59 (24.89) | 88 (59.46) |  |  |
| Swallowing Function |  |  |  | χ^2^=30.39 | ＜0.001 |
| Normal | 351 (91.17) | 231 (97.47) | 120 (81.08) |  |  |
| Abnormal | 34 (8.83) | 6 (2.53) | 28 (18.92) |  |  |
| Type of Hip Fracture |  |  |  | χ^2^=1.08 | 0.30 |
| Femoral Neck Fracture | 287 (74.55) | 181 (76.37) | 106 (71.62) |  |  |
| Intertrochanteric/Subtrochanteric Fracture | 98 (25.45) | 56 (23.63) | 42 (28.38) |  |  |
| Time from Fracture to Surgery (h) | 153 (107, 253) | 133 (96, 238) | 165.50 (120.75, 276.75) | Z=﹣2.80 | 0.01 |

**Note.** Median (P25, P75); n (%); chi-square test (χ^2^); Mann-Whitney U test (Z); Fisher's exact test (-); Transient Ischemic Attack (TIA); Chronic Obstructive Pulmonary Disease (COPD); age-adjusted Charlson Comorbidity Index (aCCI).

**Table S9.** Univariate Analysis of Blood Biochemical Profiles for Malnutrition in the Training Set

| **Variables** | **Training Set（N=385）** | **Well-Nourished（N=237）** | **Malnourished（N=148）** | **Statistics** | **P value** |
| --- | --- | --- | --- | --- | --- |
| HGB（g/L） | 119.30±19.30 | 122.64±18.09 | 113.95±20.03 | t=4.40 | **＜0.001** |
| WBC（10^9^/L） | 7.44 (6.00, 8.84) | 7.33 (5.73, 8.63) | 7.72 (6.35, 9.30) | Z=﹣2.203 | **0.03** |
| LYM（10^9^/L） | 1.05 (0.78, 1.34) | 1.15 (0.87, 1.46) | 0.91 (0.68, 1.17) | Z=﹣5.13 | **＜0.001** |
| NEUT（10^9^/L） | 5.57 (4.21, 7.10) | 5.34 (4.00, 6.61) | 6.12 (4.51, 7.69) | Z=﹣3.14 | **0.002** |
| PLT（10^9^/L） | 191.00 (153.00, 234.00) | 193.00 (161.00, 236.00) | 183.00 (142.75, 226.75) | Z=﹣2.31 | **0.02** |
| NLR | 5.11 (3.39, 8.29) | 4.34 (3.02, 7.18) | 6.76 (4.18, 9.56) | Z=﹣5.16 | **＜0.001** |
| PLR | 175.68 (134.19, 246.59) | 166.32 (128.97, 233.33) | 188.81 (146.73, 267.32) | Z=﹣2.96 | **0.003** |
| CRP（mg/L） | 19.33 (8.56, 40.30) | 15.52 (7.52, 33.70) | 31.07 (12.50, 55.36) | Z=﹣4.82 | **＜0.001** |
| TP（g/L） | 63.52±7.10 | 65.18±6.49 | 60.86±7.25 | t=6.07 | **＜0.001** |
| ALB（g/L） | 37.37±4.91 | 39.17±4.20 | 34.49±4.60 | t=10.25 | **＜0.001** |
| PA（mg/L） | 163.00 (130.00, 197.00) | 176.50 (142.60, 210.00) | 140.50 (114.00, 170.32) | Z=﹣6.59 | **＜0.001** |
| GLU（mmol/L） | 5.62 (4.94, 6.62) | 5.57 (4.93, 6.41) | 5.76 (4.94, 7.08) | Z=﹣1.34 | 0.18 |
| Ca^2+^（mmol/L） | 2.15 (2.05, 2.24) | 2.17 (2.09, 2.25) | 2.09 (2.01, 2.19) | Z=﹣5.35 | **＜0.001** |
| Cr（umol/L） | 57.00 (47.00, 70.00) | 57.00 (47.10, 68.00) | 56.00 (47.00, 74.78) | Z=﹣0.59 | 0.56 |
| BUN（mmol/L） | 6.00 (4.80, 7.70) | 5.80 (4.80, 7.40) | 6.40 (4.79, 8.36) | Z=﹣2.35 | **0.02** |
| ALT（U/L） | 15.00 (11.50, 20.10) | 16.00 (12.00, 21.00) | 14.00 (11.00, 19.10) | Z=﹣2.38 | **0.02** |
| AST（U/L） | 20.00 (17.00, 25.00) | 20.00 (17.00, 24.80) | 20.00 (16.30, 26.00) | Z=﹣0.39 | 0.69 |

**Note.** Mean +/- SD; Median (P25, P75); n (%); Student’s t-test (t); chi-square test (χ^2^); Mann-Whitney U test (Z); Haemoglobin (HGB); White Blood Cell Count (WBC); Lymphocyte Count (LYM); Neutrophil Count (NEUT); Platelet Count (PLT); Neutrophil-to-Lymphocyte Ratio (NLR); Platelet-to-Lymphocyte Ratio (PLR); C-Reactive Protein (CRP); Total Protein (TP); Albumin (ALB); Prealbumin (PA); Glucose (GLU); Calcium (Ca²⁺); Creatinine (Cr); Blood Urea Nitrogen (BUN); Alanine Aminotransferase (ALT); Aspartate Aminotransferase (AST).

**Table S10.** Univariate Analysis of Surgery-Related Factors for Malnutrition in the Training Set

| **Variables** | **Training Set（N=385）** | **Well-Nourished（N=237）** | **Malnourished（N=148）** | **Statistics** | **P value** |
| --- | --- | --- | --- | --- | --- |
| Type of Surgery |  |  |  | χ^2^=9.00 | 0.01 |
| Total Hip Arthroplasty | 229 (59.48) | 150 (63.29) | 79 (53.38) |  |  |
| Partial Hip Arthroplasty | 55 (14.29) | 24 (10.13) | 31 (20.95) |  |  |
| Open Reduction and Internal Fixation | 101 (26.23) | 63 (26.58) | 38 (25.68) |  |  |
| Type of Anesthesia |  |  |  | χ^2^=2.10 | 0.15 |
| General Anesthesia | 318 (82.60) | 201 (84.81) | 117 (79.05) |  |  |
| Neuraxial Anesthesia | 67 (17.40) | 36 (15.19) | 31 (20.95) |  |  |
| Duration of Surgery (min) | 100 (80, 125) | 100 (80, 120) | 102 (78.75, 125) | Z=﹣0.31 | 0.76 |
| Duration of Anesthesia (min) | 130 (105, 155) | 130 (105, 150) | 130 (100, 160) | Z=﹣0.94 | 0.35 |
| Intraoperative Blood Loss (mL) | 200 (100, 300) | 150 (100, 250) | 200 (100, 300) | Z=﹣1.50 | 0.13 |
| Intraoperative Blood Transfusion |  |  |  | χ^2^=12.57 | ＜0.001 |
| No | 274 (71.17) | 184 (77.64) | 90 (60.81) |  |  |
| Yes | 111 (28.83) | 53 (22.36) | 58 (39.19) |  |  |
| ASA Physical Status Classification |  |  |  | χ^2^=99.58 | ＜0.001 |
| ≤ II | 140 (36.36) | 132 (55.70) | 8 (5.41) |  |  |
| II | 245 (63.64) | 105 (44.30) | 140 (94.59) |  |  |
| Preoperative Fasting Time for Clear Fluids (h) | 6 (6, 8) | 6 (6, 8) | 6 (6, 6) | Z=﹣2.17 | 0.03 |
| Preoperative Fasting Time for Solids (h) | 8 (8, 10) | 8 (8, 10) | 8 (8, 8) | Z=﹣1.16 | 0.25 |
| Postoperative Drainage Volume (mL) | 75 (40, 125) | 65 (35, 115) | 97.50 (55, 146.25) | Z=﹣4.21 | ＜0.001 |
| Postoperative Bed Rest Duration (d) | 5 (4, 7) | 4 (3, 5) | 7 (5, 8) | Z=﹣11.68 | ＜0.001 |

**Note.** Median (P25, P75); n (%); chi-square test (χ^2^); Mann-Whitney U test (Z); American Society of Anesthesiologists (ASA).

**Table S11.** Univariate Analysis of Medication Use and Diet-Related Factors for Malnutrition in the Training Set

| **Variables** | **Training Set（N=385）** | **Well-Nourished（N=237）** | **Malnourished（N=148）** | **Statistics** | **P value** |
| --- | --- | --- | --- | --- | --- |
| Antihypertensive Drugs |  |  |  | χ^2^=2.46 | 0.12 |
| No | 157 (40.78) | 104 (43.88) | 53 (35.81) |  |  |
| Yes | 228 (59.22) | 133 (56.12) | 95 (64.19) |  |  |
| Lipid-Lowering Drugs |  |  |  | χ^2^=1.97 | 0.16 |
| No | 353 (91.69) | 221 (93.25) | 132 (89.19) |  |  |
| Yes | 32 (8.31) | 16 (6.75) | 16 (10.81) |  |  |
| Hypoglycemic Drugs |  |  |  | χ^2^=4.26 | 0.04 |
| No | 273 (70.91) | 177 (74.68) | 96 (64.86) |  |  |
| Yes | 112 (29.09) | 60 (25.32) | 52 (35.14) |  |  |
| Diuretics |  |  |  | χ^2^=19.88 | ＜0.001 |
| No | 338 (87.79) | 222 (93.67) | 116 (78.38) |  |  |
| Yes | 47 (12.21) | 15 (6.33) | 32 (21.62) |  |  |
| Hormone Drugs |  |  |  | χ^2^=8.88 | 0.003 |
| No | 365 (94.81) | 231 (97.47) | 134 (90.54) |  |  |
| Yes | 20 (5.19) | 6 (2.53) | 14 (9.46) |  |  |
| Acid-Suppressing |  |  |  | χ^2^=63.71 | ＜0.001 |
| No | 203 (52.73) | 163 (68.78) | 40 (27.03) |  |  |
| Yes | 182 (47.27) | 74 (31.22) | 108 (72.97) |  |  |
| Antipsychotic Drugs |  |  |  | χ^2^=15.83 | ＜0.001 |
| No | 339 (88.05) | 221 (93.25) | 118 (79.73) |  |  |
| Yes | 46 (11.95) | 16 (6.75) | 30 (20.27) |  |  |
| Nutritional Supplements |  |  |  | χ^2^=1.85 | 0.17 |
| No | 334 (86.75) | 210 (88.61) | 124 (83.78) |  |  |
| Yes | 51 (13.25) | 27 (11.39) | 24 (16.22) |  |  |
| Daily Protein Intake (g) | 39.00 (34.30, 44.30) | 41.80 (38.40, 46.30) | 34.20 (30.80, 38.25) | Z=﹣10.83 | ＜0.001 |
| Daily Carbohydrate Intake (g) | 137.20 (130.80, 143.20) | 140.60 (135.80, 146.20) | 131.35 (122.50, 135.72) | Z=﹣10.47 | ＜0.001 |
| Daily Dietary Fiber Intake (g) | 26.20 (21.50, 29.40) | 28.40 (25.10, 31.30) | 22.35 (19.17, 25.70) | Z=﹣9.25 | ＜0.001 |
| Daily Fat Intake (g) | 26.60 (22.60, 30.40) | 28.60 (25.50, 31.20) | 22.90 (20.98, 27.30) | Z=﹣8.26 | ＜0.001 |
| Daily Energy Intake (kcal) | 1369.00 (1284.00, 1438.00) | 1413.00 (1351.00, 1475.00) | 1286.50 (1185.25, 1340.00) | Z=﹣10.88 | ＜0.001 |

**Note.** Median (P25, P75); n (%); chi-square test (χ^2^); Mann-Whitney U test (Z).

**Table S12.** Results of Backward Stepwise Regression Analysis (AIC-based step)

| **Step** | **Variable Removed** | **Df** | **Deviance** | **Resid. Df** | **Resid. Dev** | **AIC** |
| --- | --- | --- | --- | --- | --- | --- |
| 1 | - | - | - | 374 | 307.48 | 329.48 |
| 2 | Sleep Wake Disorders | 1 | 0.575 | 375 | 308.06 | 328.06 |
| 3 | Oral Health | 1 | 0.759 | 376 | 308.82 | 326.82 |

**Note.** Degrees of Freedom (Df); Residual Degrees of freedom (Resid. Df); Residual Deviance (Resid. Dev); Akaike Information Criterion (AIC).

**Table S13.** Settings of Model Parameter Combinations

| **Model** | **Hyperparameter** | **Tuned/Fixed** | **Search Range / Options** | **Final Selected Value** |
| --- | --- | --- | --- | --- |
| LR | family | Fixed | - | binomial |
|  | direction | Fixed | - | backward |
| RF | ntree | Fixed | - | 50 |
|  | mtry | Tuned | 1–8 | 1 |
|  | nodesize | Tuned | 1–10 | 5 |
|  | replace | Fixed | - | TRUE |
|  | importance | Fixed | - | TRUE |
|  | nPerm | Fixed | - | 1000 |
| SVM | kernel | Fixed | - | radial |
|  | cost | Tuned | 0.1, 1, 10 | 1 |
|  | gamma | Tuned | 0.01, 0.1, 1 | 0.1 |
|  | epsilon | Fixed | - | - |
|  | scale | Fixed | - | TRUE |
|  | probability | Fixed | - | TRUE |
| XGBoost | nrounds | Tuned | 50–200 | 100 |
|  | objective | Fixed | - | binary:logistic |
|  | eval_metric | Fixed | - | logloss |
|  | eta | Tuned | 0.01, 0.1, 0.3 | 0.1 |
|  | max_depth | Tuned | 2–6 | 2 |
|  | subsample | Tuned | 0.6, 0.8, 1.0 | 0.8 |
|  | min_child_weight | Tuned | 1–5 | 1 |
|  | colsample_bytree | Tuned | 0.6, 0.8, 1.0 | 0.8 |
|  | lambda | Fixed | - | 0 |
|  | alpha | Fixed | - | 0 |
|  | early_stopping | Fixed | - | 10 |
| ANN | size | Tuned | 3–15 | 10 |
|  | decay | Fixed | - | 0.1 |
|  | maxit | Fixed | - | 200 |
|  | linout | Fixed | - | FALSE |
|  | activation | Fixed | - | sigmoid |
|  | skip | Fixed | - | FALSE |

**Note.** Tuning strategy: A 5-fold cross-validated grid search was conducted exclusively on the training set, with AUC maximization as the optimization objective. In the Search Range column, comma-separated values indicate discrete candidate values, whereas values connected by an en dash represent a continuous integer range.

**Table S14.** Training Set Performance: Comparison of Model Discriminative Performance Before and After Removal of GLIM-Overlapping Variables

| Model | AUC (Original Model) | AUC (Reduced Model) | ΔAUC | P value |
| --- | --- | --- | --- | --- |
| XGBoost | 0.94 (95% CI: 0.92–0.96) | 0.93 (95% CI: 0.90–0.95) | –0.02 | 0.06 |
| RF | 0.94 (95% CI: 0.91–0.96) | 0.92 (95% CI: 0.89–0.95) | –0.02 | 0.03 |
| ANN | 0.90 (95% CI: 0.87–0.93) | 0.85 (95% CI: 0.81–0.89) | –0.06 | 0.0001 |
| SVM | 0.90 (95% CI: 0.86–0.93) | 0.88 (95% CI: 0.84–0.91) | –0.02 | 0.06 |
| LR | 0.89 (95% CI: 0.86–0.92) | 0.88 (95% CI: 0.85–0.92) | –0.01 | 0.10 |

**Table S15.** Training Set Performance: Comparison of Model Discriminative Performance Before and After Removal of GLIM-Overlapping Variables

| Model | AUC (Original Model) | AUC (Reduced Model) | ΔAUC | P value |
| --- | --- | --- | --- | --- |
| XGBoost | 0.85 (95% CI: 0.79–0.91) | 0.83 (95% CI: 0.76–0.90) | –0.02 | 0.13 |
| RF | 0.86 (95% CI: 0.79–0.92) | 0.85 (95% CI: 0.78–0.91) | –0.01 | 0.46 |
| ANN | 0.84 (95% CI: 0.77–0.90) | 0.77 (95% CI: 0.69–0.84) | –0.07 | 0.01 |
| SVM | 0.84 (95% CI: 0.77–0.90) | 0.82 (95% CI: 0.75–0.89) | –0.02 | 0.31 |
| LR | 0.86 (95% CI: 0.80–0.92) | 0.85 (95% CI: 0.79–0.91) | –0.01 | 0.30 |
